# Expression of the TEL-Syk Fusion Protein in Hematopoietic Stem Cells Leads to Rapidly Fatal Myelofibrosis in Mice

**DOI:** 10.1371/journal.pone.0077542

**Published:** 2013-10-08

**Authors:** Michelle T. Graham, Clare L. Abram, Yongmei Hu, Clifford A. Lowell

**Affiliations:** Department of Laboratory Medicine, University of California San Francisco, San Francisco, California, United States of America; University of Torino, Italy

## Abstract

The TEL-Syk fusion protein was isolated from a patient with myelodysplasia with megakaryocyte blasts. Expression of TEL-Syk transforms interleukin-3 (IL-3)-dependent Ba/F3 cells *in vitro* by deregulating STAT5-mediated signal transduction pathways. *In vivo*, TEL-Syk expression in pre-B cells blocks B cell differentiation, leading to lymphoid leukemia. Here, we demonstrate that TEL-Syk introduced into fetal liver hematopoietic cells, which are then adoptively transferred into lethally irradiated recipients, leads to an aggressive myelodysplasia with myelofibrosis that is lethal in mice by 60–75 days. Expression of TEL-Syk induces a short-lived myeloexpansion that is rapidly followed by bone marrow failure and extreme splenic/hepatic fibrosis accompanied by extensive apoptosis. The disease is dependent on Syk kinase activity. Analysis of serum from TEL-Syk mice reveals an inflammatory cytokine signature reminiscent of that found in the sera from patients and mouse models of myeloproliferative neoplasms. TEL-Syk expressing cells showed constitutive STAT5 phosphorylation, which was resistant to JAK inhibition, consistent with deregulated cytokine signaling. These data indicate that expression of TEL-Syk in fetal liver hematopoietic cells results in JAK-independent STAT5 phosphorylation ultimately leading to a uniquely aggressive and lethal form of myelofibrosis.

## Introduction

Myeloproliferative neoplasms (MPNs) encompass a number of myeloid malignancies that arise from clonal hematopoietic stem cells and progenitors. MPNs are characterized by differential myeloid cell proliferation that manifest as eight different forms, with Philadelphia chromosome (encoding the BCR-ABL fusion protein) positive chronic myeloid leukemia (CML) and the BCR-ABL negative diseases polycythemia vera (PV), essential thrombocytopenia (ET), and primary myelofibrosis (PMF) being the most common [1]. Deregulated activation of tyrosine kinases, either through point mutations or generation of fusion proteins, is common to many MPNs [2]. *JAK2V617F* is found in >50% of PMF patients and leads to progressive anemia, splenomegaly, myelo-expansion, and fibrosis of the bone marrow. This mutation disrupts autoinhibition of JAK2 and drives deregulated signal transduction downstream of multiple cytokine receptors [3]. Other examples of deregulated tyrosine kinases fusion genes which are found in myeloid malignancies include *TEL-ABL*, *TEL-JAK2, FLT3/ITD* in acute myeloid leukemia (AML), *ETV6-PDGFRB* (TEL-PDGFRB) in chronic myelomonocytic leukemia (CMML), and *FIP1L-PDGFRA* in chronic eosinophilic leukemia (CEL) [4], [5]. TEL-PDGFRB, TEL-JAK2 and TEL-ABL proteins are constitutively active tyrosine kinases and lead to deregulated signaling through TEL-induced oligomerization [6].

Spleen tyrosine kinase, or Syk, is a non-receptor tyrosine kinase that signals downstream of immunoreceptors and integrins in hematopoietic cells [7]. Syk modulates cell survival in various human hematopoietic malignancies; overexpression of Syk promotes survival of non-Hodgkin’s lymphoma cell lines [8] and limits differentiation of AML cell lines [9].

Fusion proteins involving Syk kinase have been identified in two types of hematopoietic malignancies; T-cell lymphoma [10] and myleodysplastic syndrome (MDS) [6]. In T-cell lymphoma, Syk is fused to the Tec family tyrosine kinase ITK [11], forming a protein consisting of the PH domain of ITK fused to the kinase domain of Syk. When expressed in mouse hematopoietic stem cells, this protein produces a T-cell lymphoma, phenocopying the human disease [12]. Ablation of either the PH domain of ITK or the kinase domain of Syk blocks transformation *in vitro* [13].

The TEL-Syk fusion protein was first isolated from a patient with MDS accompanied by megakarocyte blasts [6]. *TEL-Syk* consists of the N-terminal pointed (PNT) domain of TEL fused to the kinase domain of Syk. TEL, also known as ETV6, is a transcriptional repressor involved in establishing definitive hematopoiesis [14], [15]. As stated above, TEL has been implicated in a number of hematological malignancies, usually as a result of its fusion to various tyrosine kinases [15]. Expression of the TEL-Syk fusion protein confers growth factor independence on Ba/F3 cells, while expression in primary pre-B cells leads to lymphoid leukemia in mice [6], [16]. In Ba/F3 cells, expression of TEL-Syk leads to the activation of numerous signaling pathways, including the PI3 kinase/AKT and MAP kinase pathways, as well as activation of cytokine signaling pathways downstream of JAK2 (through phosphorylation of STAT5) [17]. In pre-B cells, TEL-Syk expression leads to a general increase in tyrosine phosphorylation [16]. Therefore, the question remains whether expression of TEL-Syk in mouse hematopoietic stem cells will induce a myeloid malignancy resembling the human disease from which the fusion protein was identified. To address this question, we retrovirally transduced TEL-Syk into mouse fetal liver cells then studied the growth of these cells in culture or following adoptive transfer into irradiated recipient mice. TEL-Syk expression in fetal liver hematopoietic cells lead to an aggressive form of myelodysplasia accompanied by fibrosis that was dependent on the kinase domain of Syk inducing STAT5 phosphorylation despite JAK2 inhibition.

## Materials and Methods

### Ethics Statement

This study was carried out in strict accordance with the recommendations in the Guide for the Care and Use of Laboratory Animals of the National Institutes of Health. The protocol was approved by the UCSF Institutional Animal Care and Use Committee (approval number: AN090458-01). All surgery was performed under isoflurane anesthesia, and all efforts were made to minimize suffering.

### Mice

BALB/c (CD45.2) mice were purchased from Taconic Laboratories. Mice were housed in the UCSF rodent barrier facility, under the care of UCSF Laboratory for Animal Resources Center (LARC) staff, which is an AAALAC accredited specific pathogen free facility. Animals were monitored for development of myelodysplasia, following transfer of retrovirally transduced progenitors (see below) daily by LARC staff and by laboratory personnel. Any animals showing evidence of illness, as defined by ruffled fur, poor motility, apparent body weight loss, skin or abdominal swelling or who had a body condition score of 2 or less, were considered to have reached the humane endpoint of experiment and were removed from the study for euthanasia. Mice were also monitored every two weeks by serial blood sampling to look for evidence of myelodysplasia. Euthanasia was conducted by CO_2_ inhalation in small chambers within the barrier facility followed by cervical dislocation to ensure death. Tissues were removed for study as described below. Induction of myleodysplasia by retroviral transduction was done in groups of 3–5 mice per experimental retrovirus used. Each experiment usually had 4 different groups (including a control retrovirus cohort).

### Reagents

Human TEL-Syk was kindly provided by Dr. Hassan Jumaa [16]. Murine IL-6, IL-3, SCF, IL-11, GM-CSF (all from Peprotech) and Flt3L (R&D Systems) were used to culture primary fetal liver hematopoietic cells subsequently used for CFU-assays, chimera formation, and *in vitro* cytokine stimulation. For JAK inhibition, we used the JAK inhibitor 1 (EMD Millipore). Anti-Syk antibodies (Cell Signaling), anti-NTAL (Cell Signaling), p-Tyr (4G10), phospho-STAT5 and total STAT5 (14H2 and 3H7, Cell Signaling), anti-Erk1 and Erk2 (Santa Cruz Biotech), anti-GFP (Novus), and fluorescently labeled goat anti-mouse or rabbit secondary antibodies were used (LI-COR Biosciences) for immunoblot analysis. Immunoblots were imaged using the Odyssey Infrared Imaging system (LI-COR Biosciences). Antibodies used for flow cytometry included mIgG (Sigma-Aldrich), anti-CD16/CD32 (2.4G2; UCSF), anti-CD11b (M1/70), anti-Ly6G (1A8), anti-TCRβ (H57-597), anti-CD19 (ID3), anti-NKp46 (9E2), anti-Siglec-F (E50-2440), anti-CD71 (C2F2), anti-TER-119 (TER-119) phospho-STAT5-Alexa Fluor 647 (47) from (BD Biosciences); F4/80 (CI:A3-1, Serotec), anti-GFP Biotin (5F12.4, eBiosciences) and streptavidin Pacific Orange (Invitrogen).

### Biochemical analysis

For NTAL phosphorylation, HEK293T cells were washed in ice-cold PBS containing 1 mM Na _3_VO_4_ and lysed in RIPA buffer (20 mM Tris-HCl pH 7.5, 150 mM NaCl, 1% (vol/vol) Triton X-100, 1% (wt./vol) sodium deoxycholate, 0.1% (wt./vol) SDS) containing 1 mM Na _3_VO_4_, 50 mM NaF, 2 mM EDTA, 1 mM Pefabloc, 10 µg/ml of leupeptin, 2 µg/ml of aprotinin, 1 mM dithiothreitol, 1 µg/ml of pepstatin and 1 mM di-isopropyl fluorophosphate. Following addition of sample buffer (0.25 M Tris-HCl pH 6.8, 2% SDS, 75 mM DTT,10% glycerol, 0.0125% bromo-phenol blue) and boiling for 10 minutes at 95°C, lysates were separated by SDS-PAGE, transferred to Immobilon-F PVDF membrane (Millipore) and probed with antibodies as indicated.

For the *in vitro* kinase assay, lysates from transfected HEK 293T cells were immunoprecipitated with anti-Syk antibody and incubated with protein-A/G Plus agarose beads (Santa Cruz Biotech). Beads were washed in kinase buffer (20 mM HEPES pH 7.4, 10 mM MgCl_2_, 2 mM MnCl_2_, 1 mM dithiothreitol) and then incubated in kinase buffer containing 5 µCi γ-ATP (PerkinElmer) for 20 minutes at 25°C. The reaction was stopped by addition of sample buffer and separated by SDS-PAGE. Gels were fixed and stained with Coomasie blue then dried and exposed to BioMax (Kodak) film.

For analysis of phospho-STAT5 and total STAT5, GFP^+^ infected fetal liver cells were collected and washed three times in cold PBS. Cells were lysed directly in sample buffer at 5 x10^6^ cells/mL and analyzed by SDS-PAGE followed by immunoblotting.

For analysis of total phospho-tyrosine and Syk in tissues from mice reconstituted with vector, TEL-Syk KD or TEL-Syk transduced fetal liver cells, 5 x10^5^ splenocytes or bone marrow cells were washed twice in PBS and lysed in sample buffer. Cell lysates were analyzed by SDS-PAGE followed by immunoblotting.

### Retroviral infection of fetal liver cells

Retroviral supernatants were generated by transient transfection of HEK-293T cells as previously described [18]. HEK 293T cells were cultured in Dulbecco’s Modified Eagle’s Medium (DMEM) supplemented with 10% fetal bovine serum (FBS) (vol/vol), 100 µg/mL penicillin and 100 U/mL streptomycin. Mouse Syk, human TEL-Syk or human TEL-Syk kinase dead (KD) were cloned into the retroviral expression vector pMIG-W, which also encodes an internal IRES-GFP for marking of infected cells. TEL-Syk KD was generated using the Quikchange site-directed mutagenesis kit (Stratagene) using primers (Sigma-Aldrich) to introduce the K473A mutation as previously described [16].

Fetal liver cells from day 16-19 embryos were isolated and cultured overnight in Iscove’s Modified Dulbecco’s Medium (IMDM) containing 15% (vol/vol) FBS and cytokines (10 ng/ml mouse IL-6, 20 ng/ml murine IL-3, 100 ng/ml mouse SCF). For retroviral infection, 5-7 x 10^6^ isolated fetal liver cells were spin-infected at 2000 rpm for 1 hour at 25°C in retroviral supernatants supplemented with 15% FBS, 8 µg/mL polybrene (Sigma), and cytokines, and incubated at 37°C overnight. Cells were washed the next day and placed in IMDM containing 15% (vol/vol) FBS and cytokines. Retroviral infection efficiency was determined by flow cytometry using GFP expression as a marker.

### CFU Assays

**Figure 1:**
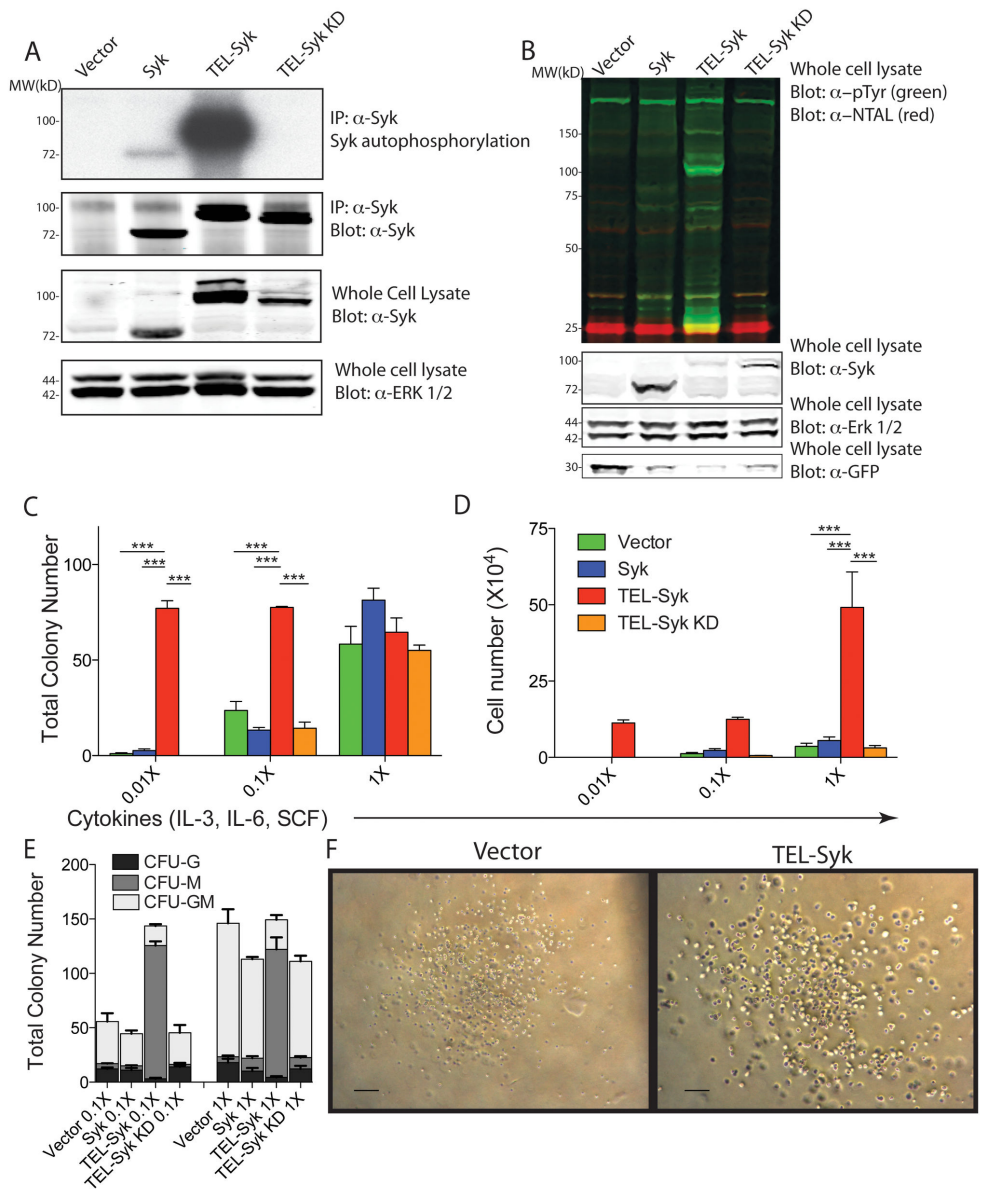
TEL-Syk is constitutively active and conferred hypersensitivity to low cytokine levels. (A) Autophosphorylation of TEL-Syk. HEK293T cells were transiently transfected with empty vector, Syk, TEL-Syk, and TEL-Syk KD. For the *in* *vitro* kinase assay, cell lysates were immunoprecipitated with anti-Syk then incubated with P^32^ labeled ATP. Immunoblots demonstrating levels of Syk, TEL-Syk and TEL-Syk KD are shown below with Erk1/2 as a loading control. (B) Phosphorylation of NTAL and other cellular proteins by TEL-Syk. HEK293T cells were transiently co-transfected with NTAL plus empty vector, Syk, TEL-Syk, or TEL-Syk KD. Whole cell lysates were immunoblotted as indicated. Immunoblots are representative of three independent experiments. Note that expression of TEL-Syk was frequently lower than Syk or TEL-Syk KD in many experiments, likely because TEL-Syk is somewhat toxic to cells. Hence the TEL-Syk band is weaker in the whole cell lysate panel, despite the fact that overall tyrosine phosphorylation is much higher. (C-D) Expression of TEL-Syk leads to growth factor hypersensitivity in fetal liver hematopoietic progenitors. Syk-deficient fetal livers were transduced with retrovirus carrying empty vector, Syk, TEL-Syk, or TEL-Syk KD, sorted based on GFP expression then plated in methylcellulose at a range of cytokine concentrations. Total colony numbers (C), total cell numbers (D), and colony types (E) were determined 7 days after plating. (F) CFU-M brightfield images of vector and TEL-Syk at 0.1X cytokine stimulation were taken at 7 days after plating. Scale bars represent 100µm. Experiments were conducted in triplicate and data is expressed as ± SEM. Statistical significance was determined by one-way ANOVA. ***P< 0.001.

BALB/c fetal liver cells were isolated at embryonic day 16-19 and infected with retrovirus containing vector alone, Syk, TEL-Syk or TEL-Syk KD. A cytokine mix consisting of 10 ng/mL of murine IL-6, 20 ng/mL of murine IL-3, 100n g/mL of murine SCF, 10 ng/mL of IL-11, and 10 ng/mL of Flt3L in IMDM containing 15% FBS, adapted from Schubbert et al. [19], was used to culture isolated fetal liver hematopoietic cells. GFP^+^ cells were sorted using a FACS Aria (BD Biosciences) and plated at a density of 2 x 10^4^ in methylcellulose (StemCell Technologies), according to manufacturer’s directions, in 35 mm tissue culture dishes (BD Biosciences) with 1 ng/ml IL-3, 1 ng/ml IL-6, and 5 ng/ml SCF, which is indicated as the “1X” concentration in Figure 1. Cells were also plated in 10 or 100 fold dilutions of these cytokines, designated 0.1X and 0.01X respectively. Cells were cultured for 7 days, and colony numbers and frequencies were counted using a phase microscope. Images were captured using a Leica DM IL inverted contrast microscope and then processed with Leica Application Suite v3.1. For total live cells numbers, cells were harvested using IMDM containing 2 mM EDTA, stained with trypan blue. For JAK inhibition studies, cells were plated as above in the presence of 10 ng/mL GM-CSF and varying concentrations of JAK inhibitor 1. Cells were cultured for 7 days and then colonies were counted.

### Generation of radiation chimeric mice

Two to three month old BALB/c recipient mice were lethally irradiated with 1200 rads (2 doses of 600 rads separated by 3 hours). Recipients were injected retro-orbitally with 5 x 10^6^ of vector, Syk, TEL-Syk and TEL-Syk KD virally transduced cells, then placed on 120 U/mL polymyxin B/ 0.6 mg/mL neomycin antibiotics (Sigma) in their drinking water for 21 days. The degree of reconstitution was determined by following GFP expression in peripheral blood cells by flow cytometry.

### Peripheral blood analysis

Peripheral blood was collected in EDTA-containing microtainers (BD Biosciences) for complete blood count (CBC) analysis using a HEMAVET (Drew Scientific) hematology analyzer. For cell counting prior to flow cytometry, red blood cells were removed by lysis with ACK buffer (BD Biosciences), white blood cells were washed in PBS containing 2 U/mL heparin, then counted using a Nucleocounter (Chemometec). Prior to flow cytometry, white blood cells were incubated on ice with 25 µg/mL anti-CD16/CD32 and 1 mg/mL mIgG for 10 minutes then stained with a cocktail of fluorescently-labelled antibodies as indicated. Cells were washed, resuspended in 2% FBS in PBS with 5 mM EDTA containing 1 µg/mL propidium iodide (Sigma) and analyzed by flow cytometry using an LSR Fortessa (BD Biosciences). Data were analyzed using FlowJo (Tree Star Inc.).

### Peripheral blood and bone marrow smears

Peripheral blood was collected in EDTA-containing microtainers (BD Biosciences) and 5 µL of peripheral blood was placed on a Superfrost^®^ plus microscope slide (Fisher Scientific) and spread with a second slide to generate a feathered appearance. The slides were allowed to air-dry for 10 minutes and treated with Wright-Geimsa stains. For bone marrow smears, cells were flushed from one femur and tibia with PBS into a 35 mm polystyrene tissue culture dish containing PBS. Bone marrow spicules were placed in 5 µL of peripheral blood on a microscope slide. A second microscope slide was then placed on top of the peripheral blood containing bone marrow and sandwiched for 5 seconds. The slides were carefully pulled apart in one continuous motion, air-dried for 10 minutes, and treated with Wright-Geimsa stains twice. Blood and bone marrow smears were visualized using an Eclipse 80i microscope (Nikon) and images were captured using NIS-elements (Nikon).

### Cytospins

Splenocytes and bone marrow cells isolated from chimeras were resuspended in PBS at 10^3^ cells/µl. 10^5^ cells were spun onto Superfrost^®^ plus microscope slides using a Cytospin 4 (Thermo Scientific). Slides were air-dried for 10 minutes and then treated with a Differential Stain kit (Newcomer Supply) according to the manufacturer’s protocol. Cytospins were visualized under a Nikon Eclipse 80i microscope and images were captured using NIS-elements.

### Tissue staining and immunohistochemistry

Sternums, livers, and spleens were fixed in 10% vol/vol formalin and paraffin-embedded. Sections were stained with hematoxylin and eosin (H&E). Liver and spleen sections were treated with Masson’s Trichrome to indicate collagen deposition and sternum sections were stained with silver stain to indicate reticulin fibers. For enumeration of megakaryocytes, H&E sections were viewed at 100X magnification and cells were counted based on megakaryocyte morphology. For apoptosis analysis, sections were stained with anti-cleaved Caspase-3 antibodies (Cell Signaling). Slides were visualized under a Nikon Eclipse 80i microscope and images were captured using NIS-elements.

### Intracellular staining of STAT5

Fetal liver hematopoietic cells were isolated between embryonic day 14-18 and transduced with retroviral vectors, as described above. Cells were washed after retroviral transduction, placed in media containing fresh cytokines, and incubated for three additional days at 37°C. On day 3, cells were starved of cytokines for 6 hours in IMDM containing 1% FBS (vol/vol). Cells were re-stimulated with 10 ng/mL mouse IL-6, 20 ng/mL mouse IL-3, and 100 ng/mL mouse SCF for 30 minutes. Cells were washed, fixed with 2% (vol/vol) paraformaldehyde for 15 minutes at room temperature and permeabilized with ice-cold 99% (vol/vol) methanol for 10 minutes. Cells were washed three times in washing buffer (PBS containing 0.5% (wt./vol) BSA and 0.02% (wt./vol) sodium azide). Cells were incubated with 20 µg/mL anti-CD16/CD32 and 2 mg/mL mIgG for 10 minutes on ice followed by addition of fluorescently-conjugated antibodies, 10 µL Alexa-647 conjugated anti-phospho-STAT5 and 1 µg anti-GFP Biotin for 1 hour. Cells were washed twice in washing buffer and incubated with 4 µg/mL streptavidin Pacific Orange (Invitrogen) on ice for 1 hour. Cells were washed and analyzed by flow cytometry using an LSR Fortessa cell analyzer (BD Biosciences).

For JAK inhibition studies, GFP+ retrovirally infected fetal liver cells were sorted, starved for 6 hours in IMDM containing 1% (vol/vol) FBS, pretreated with JAK inhibitor 1 for 30 minutes at indicated concentrations then restimulated with 50 ng/mL of GM-CSF for 30 minutes. Cells were fixed, permeabilized, and stained as indicated above.

### RT-PCR

RNA was isolated from 1 x 10^5^ sorted GFP^+^ or GFP^-^ splenocytes or bone marrow cells from chimeric mice using the RNeasy Micro kit (Qiagen). The iScript cDNA Synthesis kit (Biorad) was used to generate cDNA. Primers previously described [6] were used to detect TEL-Syk with 30 cycles at 94°C for 40 seconds, 60°C for 1 minute, and 72°C for 1 minute. Reactions were separated by gel electrophoresis, stained with ethidium bromide and analyzed on an AlphaImager station (Alpha Innotech).

### Cytokine Analysis

Cytokine levels in peripheral blood were measured using the Cytokine Profiler Array and Angiogenesis Array (R&D systems) and pooled sera from TEL-Syk and vector chimeric mice at 30 days, 45 days, and 60 days post reconstitution. Blots were scanned and analyzed on the Kodak Digital Science™ Image Station 440CF system (Kodak).

### Statistical Analysis

Data was analyzed using Prism (Graphpad). The percentage of disease-free mice was plotted using Kaplan-Meir survival analysis and analyzed using a log-rank (Mantel-Cox) test. Differences between two groups were assessed by the unpaired *t* test; differences between three or more groups were evaluated by ANOVA, followed by Bonferroni’s Multiple Comparison post-test. Differences were considered significant when p ≤ 0.05. One-way ANOVA was performed on peripheral blood over 3 months, CFU assays, phospho-STAT5 and JAK2 inhibition studies; one-way ANOVA was performed at endpoint based on TEL-Syk morbidity for 4 genotypes.

## Results

### TEL-Syk is a hyperactive kinase that induces increased proliferative responses in hematopoietic progenitors

Previous studies have demonstrated that expression of TEL-Syk in Ba/F3 cells leads to growth factor-independent proliferation and deregulated signal transduction [6], [17]. We compared the biochemical activity of TEL-Syk and Syk expressed in HEK 293T cells. TEL-Syk was much more active in an *in vitro* kinase assay and heavily phosphorylated the downstream target NTAL (shown as a yellow band of merged phospho-tyrosine and NTAL signals) compared to Syk (Figure 1A-B). A K473A replacement rendered TEL-Syk catalytically inactive -- this TEL-Syk kinase dead (KD) mutant failed to autophosphorylate or phosphorylate NTAL.

To investigate the biological activity of Syk, TEL-Syk and TEL-Syk KD we introduced these genes into BALB/c fetal liver hematopoietic cells by retroviral infection. Using a retroviral vector containing an IRES-GFP reporter, we were able to follow the efficiency of viral transduction by flow cytometry. Transduction efficiency of fetal liver hematopoietic cells varied from an average of 10% with vector only infected cells to less than 5% of cells with Syk, TEL-Syk or TEL-Syk KD viruses (data not shown). For *ex vivo* analysis we sorted transduced cells by flow cytometry, then assessed their growth potential at various cytokine concentrations by colony forming unit (CFU) assays in methylcellulose. A cocktail of stem cell factor (SCF), IL-3, and IL-6 supported myeloid progenitor colony formation in cells expressing the retroviral vector alone, Syk or TEL-Syk KD at 1X stimulation. Ten or one hundred-fold serial dilution of the cytokine cocktail did not affect TEL-Syk colony formation, but did lead to reduced colony formation and cell expansion in vector alone, Syk or TEL-Syk KD expressing cells (Figure 1C-D). At 1X stimulation, TEL-Syk transduced fetal liver hematopoietic cells produced similar numbers of colonies, but the colony sizes were much larger and there was a ~5 fold increase in the number of cells extracted from the methylcellulose medium (Figure 1D). The distribution of colony types was also skewed in the TEL-Syk transduced cells, which showed a dramatic expansion in CFU-M (macrophage) type colonies, at both 1X and 0.1X cytokine concentrations (Figure 1E). By contrast, vector alone, Syk or TEL-Syk KD expressing cells developed mainly CFU-GM (granulocyte/macrophage) type colonies. The CFU-M colonies present in the TEL-Syk transduced samples were composed of abnormally larger cells (Figure 1F, right panel) perhaps demonstrating a more blast-like morphology. In the absence of any cytokines, no colonies were observed in any retrovirally-transduced cells. TEL-Syk expressing cells also showed increased colony sizes in GM-CSF alone CFU assays (data not shown). These data demonstrate that expression of TEL-Syk in fetal liver hematopoietic cells results in hypersensitivity to cytokine stimulation with skewing of progenitor differentiation *in vitro*.

### Adoptive transfer of TEL-Syk expressing hematopoietic progenitors leads to myeloid cell expansion and mortality

**Figure 2:**
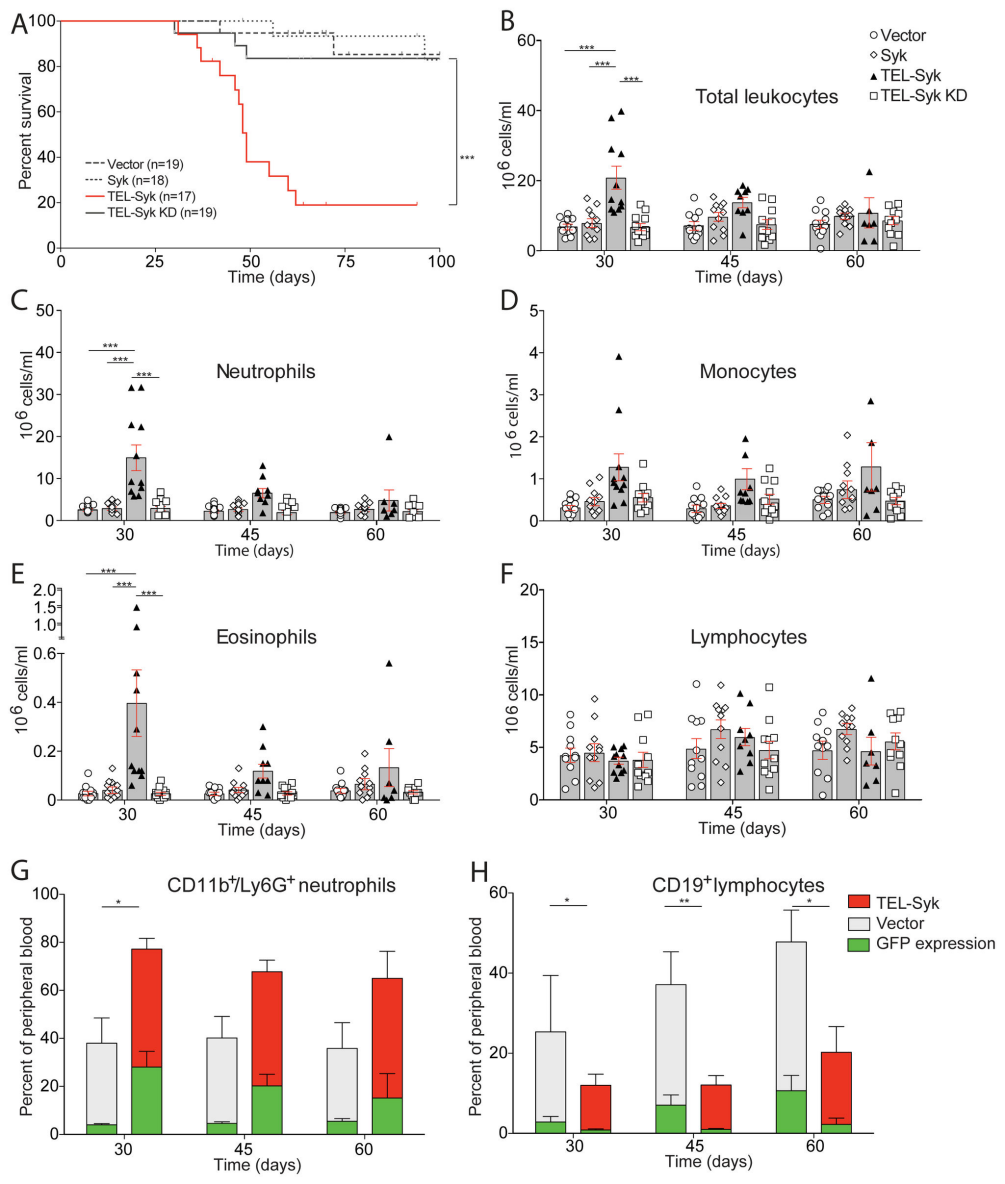
TEL-Syk expression led to myeloid cell expansion and death *in vivo*. (A) Kaplan-Meir survival curve of BALB/c recipients injected with fetal liver cells infected with retrovirus containing empty vector, Syk, TEL-Syk, or TEL-Syk KD. BALB/c fetal liver cells were spin-infected with retroviral supernatants containing the indicated viruses, then cultured for 2 days *ex* *vivo*, after which 5 x 10^6^ cells were transferred into 2-3 month old lethally irradiated recipients. Survival was followed after successful hematopoietic reconstitution (B F). CBC analysis of peripheral blood collected at the indicated number of days after transfer, including total white blood cell counts (B), and numbers of neutrophils (C), monocytes (D), eosinophils (E), and lymphocytes (F). Each data point indicates a single animal. Data are shown as mean ± SEM for vector (n=11), Syk (n=11), TEL-Syk KD (n=11), TEL-Syk (n=11 at 30 days, n= 9 at 45 days and n=7 at 60 days). (G and H) At indicated time points, peripheral blood was isolated and stained for CD11b, Ly6G and CD19 then analyzed by flow cytometry. Plots show the percentage of CD11b^+^Ly6G^+^ neutrophils (G) or CD19^+^ B cells (H), with green bars indicating the percentage of cells expressing GFP, demonstrating that they were retrovirally transduced. Data are shown as mean ± SEM for vector (n=10) or TEL-Syk (n=10) expressing mice. Statistical significance was determined by one-way ANOVA. *P< 0.05, **P<0.01, ***P< 0.001.

To examine the consequences of TEL-Syk expression in fetal liver hematopoietic cells *in vivo*, we adoptively transferred retrovirally transduced cells into irradiated recipient mice. As shown in Figure 2A, mice receiving TEL-Syk-transduced fetal liver cells had a significantly greater mortality rate post-transfer than animals receiving Syk or TEL-Syk KD transduced cells, with the majority of the mice dying within 60 days after cell transfer.

Automated complete blood count (CBC) analysis of recipient mice demonstrated leukocytosis in animals that received TEL-Syk transduced fetal liver hematopoietic cells (Figure 2B), which peaked at 30 days following cell transfer (Figure 2B). Total numbers of peripheral blood neutrophils and eosinophils were significantly increased, while monocytes were modestly increased (Figure 2C-E). Lymphocyte numbers (Figure 2F) were unchanged in all groups of mice. Robust neutrophil and eosinophil cell numbers in TEL-Syk chimeras correlated with disease severity, since mice with the highest numbers of myeloid cells at day 30 were the first to succumb (TEL-Syk chimeras that survived beyond 60 days had lower cell counts). These data strongly suggests that myelo-expansion plays a role in TEL-Syk chimeric morbidity and mortality.

We performed flow cytometry on peripheral blood samples from adoptively transferred mice (Figure S1). As seen in cohorts of mice analyzed by CBC, flow cytometric analysis showed that neutrophils, defined as Ly6G ^+^ CD11b^+^ cells were increased at all time points, most significantly at 30 and 45 days post transfer just before significant numbers of mice began to die. The numbers of B and T-lymphocytes were not significantly different from vector, Syk or TEL-Syk KD chimeric mice. Staining with anti-CD11b and anti-Siglec-F antibodies confirmed the dramatic eosinophilia in mice receiving TEL-Syk-expressing fetal liver hematopoietic cells. By contrast, mice receiving Syk or TEL-Syk KD-transduced fetal liver hematopoietic cells showed no significant hematopoietic abnormalities compared to vector alone.

Examination of peripheral blood cells for expression of the linked GFP marker in the retrovirus confirmed that expression of TEL-Syk affected only myeloid cell development and not B lymphocytes. Despite the fact that less than 5% of fetal liver hematopoietic cells were transduced with TEL-Syk, by 30 days following transfer ~30% of myeloid derived cells were GFP+ while the percentage of GFP+ B lymphocytes remained low (Figure 2G-H; note these panels are percentages while Figure S1 and Figure 2B F are absolute numbers). RT-PCR analysis confirmed that TEL-Syk was only expressed in GFP+ cells, and these cells also showed increased levels of phospho-tyrosine, in particular a 100 kD protein that likely represents TEL-Syk (Figure S2). These data indicate that expression of TEL-Syk in fetal liver cells drives a cell intrinsic expansion of myeloid lineage cells.

### TEL-Syk induces anemia and erythrodysplasia

**Figure 3:**
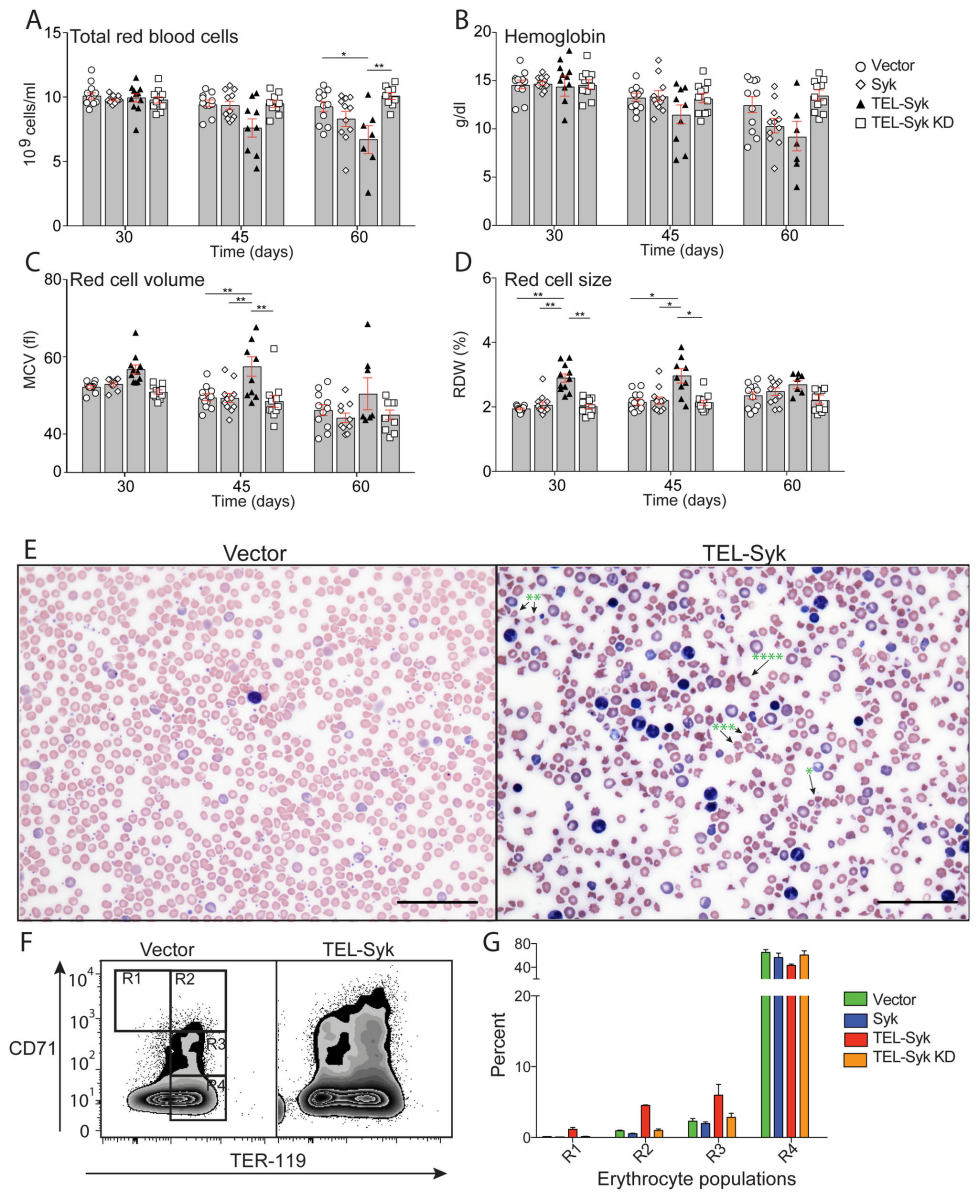
TEL-Syk induced dyserythropoiesis *in vivo*. (A-D) Peripheral blood from BALB/c recipients injected with fetal liver cells transduced with the indicated retroviruses was collected at the indicated days after transfer and assessed by CBC analysis for (A) red blood cell counts, (B) hemoglobin levels, (C) mean corpuscular volume and (D) red cell distribution width. (E) Wright-Giemsa stains of peripheral blood smears from TEL-Syk and vector mice at 45 days following fetal liver transfer. Stomatocytes, dacrocytes, acanthocytes, and spheroctyes are indicated by *, **, ***, **** respectively. (F) Flow cytometry of peripheral blood from TEL-Syk and vector expressing mice. Peripheral blood was diluted in PBS and stained for anti-CD71 and anti-TER-119. Cells stained for lineage markers Gr-1, CD11b, TCRβ, CD19, B220, and F4/80 were excluded from analysis. (E) Quantitation of erythrocyte populations R1-R5 from panel F. Scale bars correspond to 100 µm. CBC data are shown as mean ± SEM for vector (n=11), Syk (n=11), TEL-Syk KD (n=11), TEL-Syk (n=11 at 30 days, n=9 at 45 days and n=7 at 60 days) and statistical significance was determined by one-way ANOVA. *P< 0.05, **P< 0.01.

Aside from myeloid cell expansion, another hallmark feature of MDS is erythrodysplasia. Indeed, mice receiving TEL-Syk transduced fetal liver hematopoietic cells showed erythroid cell abnormalities compared to mice receiving vector, Syk or TEL-Syk KD transduced cells. Erythrocyte number and total hemoglobin levels progressively decreased in TEL-Syk chimeras (Figure 3A-B), while the remaining red blood cells showed dysplastic features as measured by increased volume (MCV) and size (RDW) (Figure 3C-D). Erythrodysplasia was readily apparent in blood smears from mice receiving TEL-Syk transduced fetal liver hematopoietic cells, as evidenced by extensive poikilocytosis, the presence of stomatocytes, dacrocytes, acanthocytes, and spheroctyes (Figure 3E and at higher magnification Figure S3). Lastly, expansion of erythroid progenitors and altered erythroid differentiation was evident in TEL-Syk expressing mice as determined by flow cytometry. Erythrocyte differentiation stages can be enumerated by Ter-119 versus CD71 staining (Figure 3F); proerythrocytes are Ter-119^med^ CD71^high^ (R1), basophilic erythroblasts are Ter-119^high^, CD71^high^ (R2), polychromatophilic erythroblasts are Ter-119^high^, CD71^med^ (R3), and orthochromatophilic erythroblasts are Ter-119^+^ (R4) [2]. TEL-Syk expressing chimeras had a 2-4 fold increase in circulating erythrocyte progenitors and a 30% reduction in mature Ter-119^+^ erythrocytes compared to control chimeras (Figure 3F-G).

### TEL-Syk chimeric mice develop hypocellular splenomegaly and extramedullary hematopoiesis

**Figure 4:**
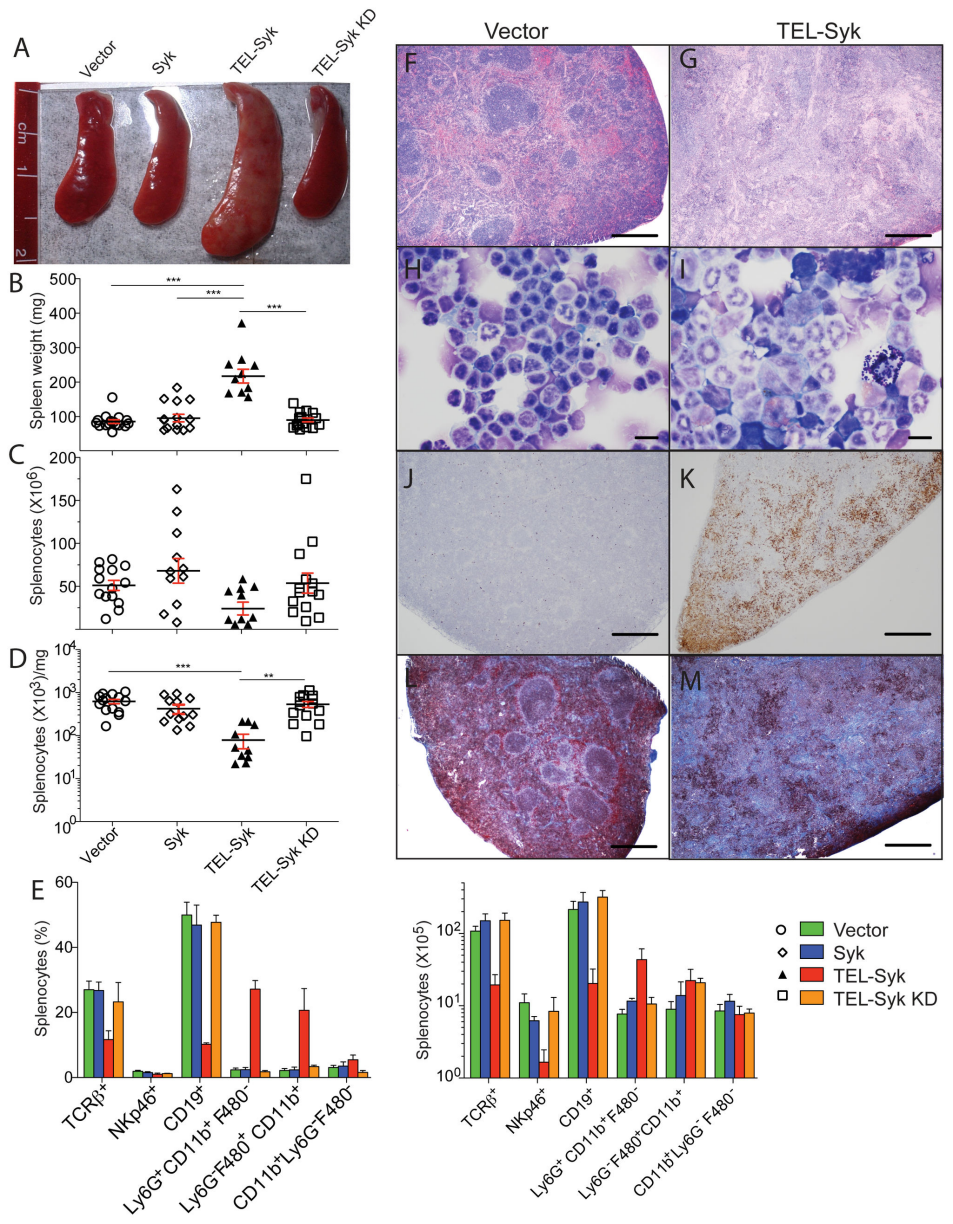
TEL-Syk chimeric mice developed hypocellular splenomegaly with accelerated cellular apoptosis. (A) Spleens from vector, Syk, TEL-Syk and TEL-Syk KD expressing mice that were sacrificed at 60 days post cell transfer. (B) Splenic weights, (C) the ratio of splenocytes per mg of spleen, and (D) total numbers of splenocytes from chimeric mice. Data are shown as mean ± SEM for vector (n=14), Syk (n=12), TEL-Syk (n=10), TEL-Syk KD (n=14). (E) Flow cytometric analysis of splenic leukocytes. Data are shown as mean ± SEM for vector, Syk, TEL-Syk, TEL-Syk KD. (F, G) H&E stained sections of spleens from vector and TEL-Syk expressing mice. (H, I) Wright-Giemsa stained cytospins of splenocytes from vector or TEL-Syk chimeras. (J, K) Spleen sections from vector and TEL-Syk expressing mice stained with anti-cleaved capase-3 antibodies to indicate apoptotic cells. (L, M) Masson’s Trichrome stained sections of spleens from vector and TEL-Syk chimeras to indicate fibrosis. Scale bars correspond to 500 µm (F, G, J-M) and 10 µm (H, I). Statistical significance was determined by one-way ANOVA. **P< 0.01, ***P< 0.001.

To evaluate the effects of TEL-Syk expression on secondary lymphoid organs, we examined the spleens of TEL-Syk chimeric mice at 60 days post fetal liver cell transfer. TEL-Syk expressing mice showed marked splenomegaly (Fig. 4A-B). Surprisingly, however, the splenomegaly was not due to increased cell numbers; in fact the TEL-Syk expressing mice had approximately 2 fold fewer splenocytes in total, resulting in nearly a 5 fold difference in the ratio of cell number to mg of spleen (Fig. 4C-D). The cells in the spleens of the TEL-Syk chimeras were predominately Ly6G ^+^ CD11b^+^ neutrophils or F4/80 ^+^ CD11b^+^ monocytes/macrophages, with a lower percentages of T and B lymphocytes, compared to the spleens of vector, Syk or TEL-Syk KD chimeras (Fig. 4E). The histology of spleens from TEL-Syk chimeras revealed disrupted follicular structures, a paucity of red pulp, islands of erythroid bodies, and large patches of connective tissue (Fig. 4F-G). At higher magnification, we observed apoptotic bodies, dysmyelopoiesis, aggregates of erythroid bodies, and eosinophilic infiltrates (Fig. S4). Cytospins of splenocytes demonstrated an increase in dysplastic neutrophils and abnormal monocytes with large disrupted nuclei in the TEL-Syk compared to vector expressing mice (Fig. 4H-I). The spleens from TEL-Syk chimeric mice showed a dramatic increase in the amount of cellular apoptosis compared to vector chimeras, as determined by immunohistochemical stains for activated caspase-3 mice (Fig. 4J-K). To examine the causes of the hypocellular splenomegaly we looked for evidence of myelofibrosis in these tissues. Using Masson’s Trichome staining, which stains collagen deposition, we found extensive fibrosis in the spleen (Figure 4L-M) sections from mice receiving TEL-Syk transduced progenitors, but little collagen deposition in vector expressing mice. Finally, the livers of the TEL-Syk chimeras showed extensive hematopoietic cell infiltration and connective tissue accumulation (Figure S5). Thus, despite the myeloid cell expansion in the peripheral blood caused by TEL-Syk expression, the spleens from these animals develop progressive hypocellularity and fibrosis associated with high levels of cell death.

### TEL-Syk chimeric mice develop bone marrow failure

**Figure 5:**
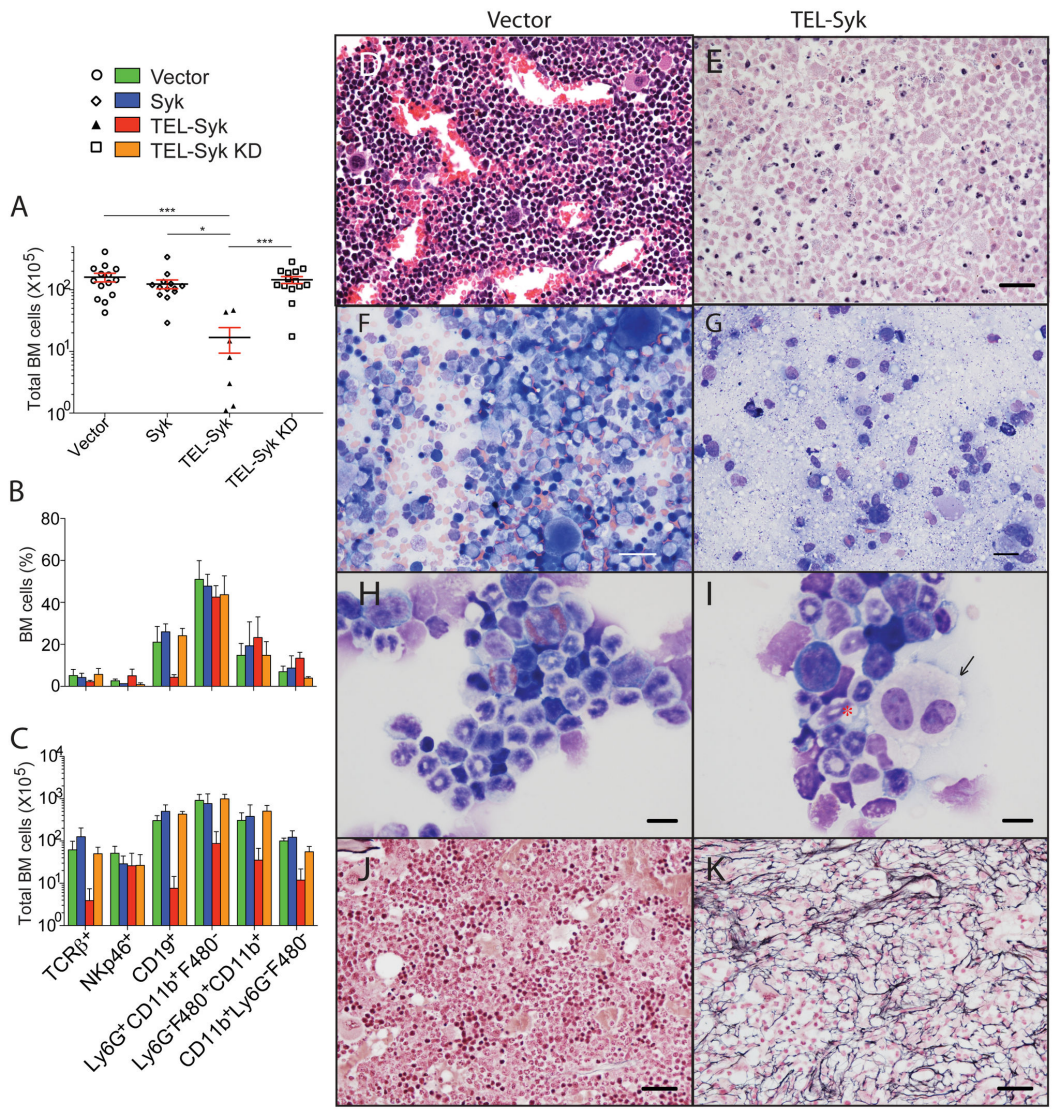
TEL-Syk chimeric mice developed bone marrow hypocellularity and fibrosis. (A) Numbers of bone marrow cells isolated from mice receiving vector, Syk, TEL-Syk KD and TEL-Syk transduced fetal liver hematopoietic cells at 60 days following cell transfer. Data are shown as mean ± SEM for vector (n=14), Syk (n=13), TEL-Syk (n=7), TEL-Syk KD (n=14). Flow cytometric analysis of (B) leukocytes percentages and (C) total leukocyte numbers in the bone marrow cells. (D, E) H&E stained sternum sections from vector and TEL-Syk expressing mice, (F, G) Wright-Giemsa staining of bone marrow smears, and (H, I) cytospins of bone marrow cells isolated from femurs and tibias of TEL-Syk and vector expressing mice at 60 days following cell transfer. Dysplastic megakaryocytes (arrow) and neutrophils (asterisk) are indicated. (J, K) Reticulin-stained sternum sections from vector and TEL-Syk expressing mice. Scale bars correspond to 30 µm (D-G, J, K) and 10 µm (H, I). Data is shown as mean ± SEM and statistical significance was determined by one-way ANOVA. *P< 0.05, ***P< 0.001.

To investigate the cause of the progressive decrease in peripheral white blood cells in mice receiving TEL-Syk transduced fetal liver cells, we examined the bone marrow of animals 60 days post cell transfer. Similar to the spleen, the bone marrow from TEL-Syk chimeric mice was markedly hypocellular compared to control mice (Figure 5A). Myeloid cells were the predominant cell type, with a relative loss of lymphoid cells in the TEL-Syk expressing mice, as determined by flow cytometry (Figure 5B-C). The bone marrow of TEL-Syk expressing mice showed extensive hypocellularity, with a general loss in diversity of hematopoietic cells (Figure 5 D-G). Bone marrow dysplasia was exemplified by the presence of mummified megakaryocytes (arrow) and dysplastic neutrophils (red asterisk) in the bone marrow of TEL-Syk chimeras (Figure 5I). We also found extensive fibrosis in sternum sections of TEL-Syk expressing mice, as determined by reticulin staining of histologic sections (Figure 5J-K). The reticulin staining was most pronounced in the hypocellular bone marrow patches of TEL-Syk chimeric mice. Thus, as in the spleen, the bone marrow of TEL-Syk chimeric mice becomes fibrotic and aplastic.

### TEL-Syk expressing mice demonstrate thrombocytopenia and low numbers of bone marrow megakaryocytes

**Figure 6:**
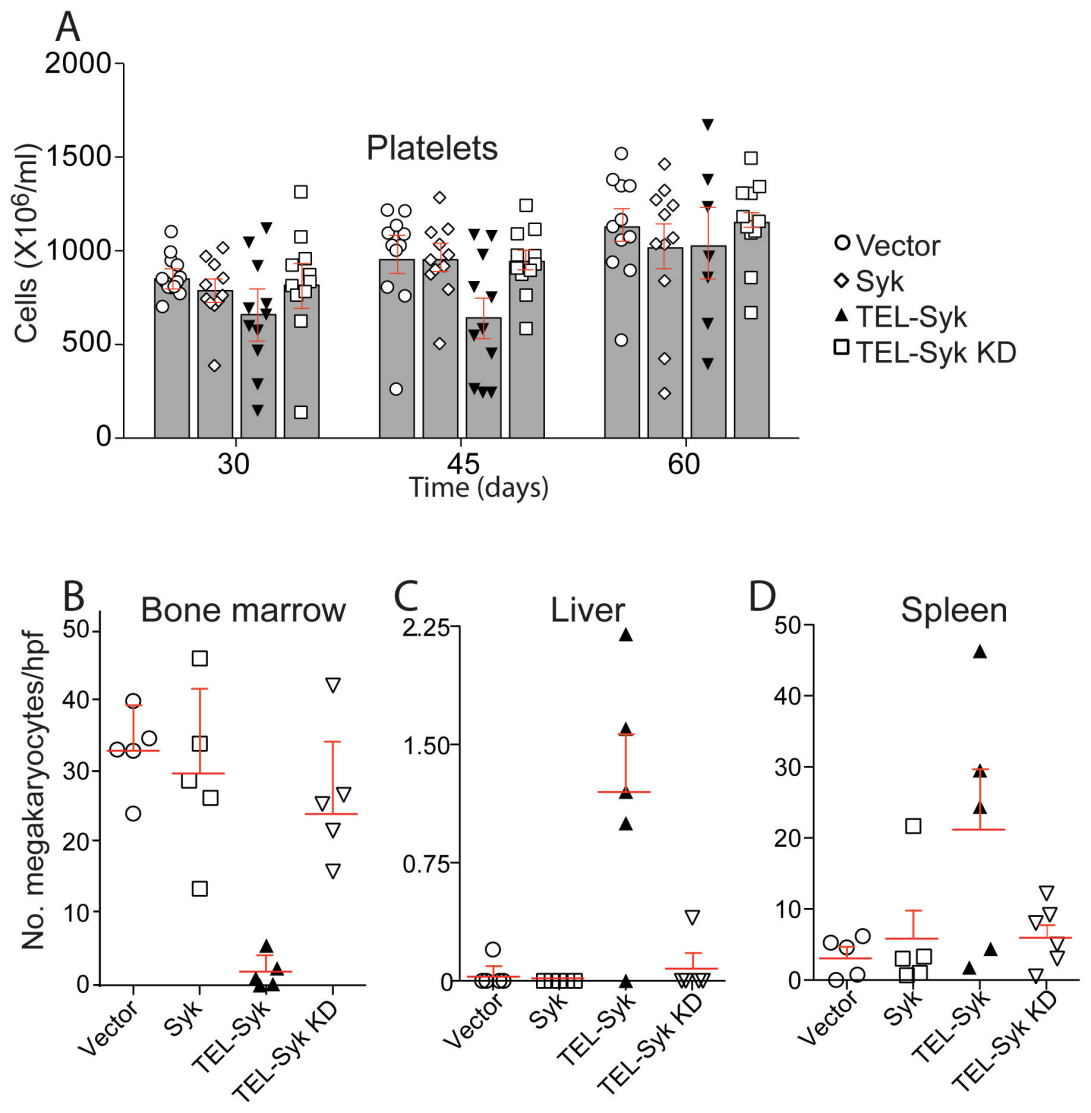
TEL-Syk expression reduced platelet numbers in the peripheral blood and megakaryocyte numbers in the bone marrow. (A) Platelet numbers were determined by CBC analysis, using a HEMAVET analyzer, of peripheral blood at the indicated days after fetal liver stem cell transfer. Due to high animal to animal variation (in part because of misinterpretation of red cell fragments by the analyzer), no statistical significance was observed (by one-way ANOVA) between groups. Data is shown as mean ± SEM for vector (n=11), Syk (n=11), TEL-Syk (n=11 at days 30 and 45, n=6 at day 60), TEL-Syk KD (n=11). Megakaryocyte numbers were quantitated per high power microscopic field in (B) bone marrow, (C) liver, and (D) spleen. An average of five fields were taken at 100X magnification of H&E sections. Data is shown as mean ± SEM for vector (n=5), Syk (n=5), TEL-Syk (n=5), TEL-Syk KD (n=5).

Similar to the progressive anemia, we noted that a number of TEL-Syk chimeric mice also manifested thrombocytopenia. The degree of thrombocytopenia varied extensively among TEL-Syk chimeras, with the lowest platelet counts seen in the animals with the most extensive disease (Figure 6A). The presence of red blood cell fragments and other cellular debris in samples from severely ill TEL-Syk chimeras may have contributed to variability in platelet counts, as this material may be confused for platelets based on light scatter properties by the automated HEMAVET counters. Thrombocytopenia was particularly evident in the peripheral blood of diseased TEL-Syk chimeras by visual examination of blood smears (Figure S3). At 60 days post stem cell transfer, megakaryocytes were virtually absent from the bone marrow of TEL-Syk expressing mice, while surviving animals showed increased megakaryocytes in the liver and spleen (again with great variation between individuals; Figure 6B-D). Though there was no evidence of obvious bleeding in TEL-Syk chimeric animals, the thrombocytopenia likely contributed to mortality in some animals.

### TEL-Syk chimeric mice manifest elevated levels of circulating inflammatory cytokines

**Figure 7:**
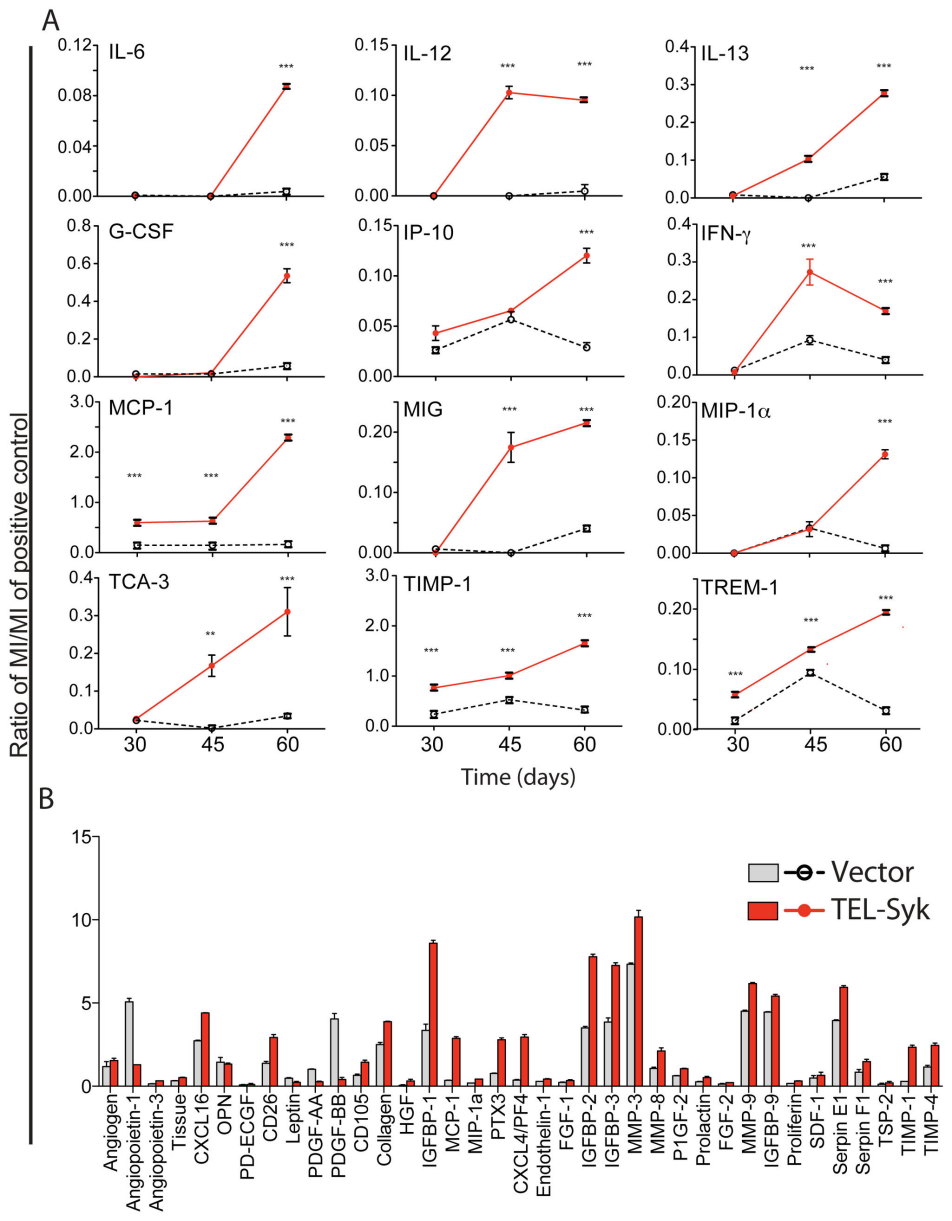
TEL-Syk expression caused temporal increases in circulating inflammatory cytokines. (A) Pooled sera collected from TEL-Syk and vector expressing mice at 30, 45 and 60 days was examined using a Cytokine Profiler Array. Cytokine levels were represented as ratio of mean intensities (MI) of the given cytokine to a positive control after background intensity subtraction. (B) Representative histograms from 30-day old pooled sera collected from TEL-Syk and vector expressing mice using the Angiogenesis Array. Data are expressed as means ± SD. Statistical significance was determined by one-way ANOVA. **P< 0.01, ***P< 0.001.

To test the hypothesis that circulating growth factors contribute to the myeloid expansion and fibrosis in TEL-Syk expressing mice, we used an immunoblot array to measure serum cytokines from TEL-Syk and vector chimeras. As shown in Figure 7A, TEL-Syk expressing mice manifested elevated levels of a number of inflammatory cytokines, growth factors, chemokines and proteases, both at 45 and 60 days following fetal liver cell transfer. IL-12, IL-13, IFN-γ, MIG/CXCL9, and TCA-3/CCL1 were robustly elevated at 30 days, while IL-6, G-CSF, IP-10/CXCL10, MCP-1/CCL2, MIP-1α/CCL3, TIMP-1 and TREM-1 became elevated at 60 days. To look for factors that may be particularly associated with fibrosis, we used an angiogenic array designed to examine a broader range of factors, assessing sera only from mice at 30 days following TEL-Syk transduction. On this array, the sera from TEL-Syk chimeric mice showed increased levels of additional chemokines, (CXCL4, CXCL16), proteases (MMP3, 8, 9), protease inhibitors (TIMP-1) and binding proteins (IGFBP1, 2, 3, 9) (Figure 7B).

### JAK inhibition failed to abolish TEL-Syk hypersensitivity and STAT5 phosphorylation

To address the mechanism by which TEL-Syk expression in hematopoietic cells drives myeloid expansion, we examined overall tyrosine phosphorylation and levels of phospho-STAT5 in cells expressing TEL-Syk. Bone marrow cells from vector, TEL-Syk, and TEL-Syk KD chimeras at 30 days following cell transfer were sorted into GFP^+^ and GFP^-^ fractions then examined by immunoblot analysis. GFP^+^ cells from TEL-Syk expressing chimeras showed higher levels of overall phospho-tyrosine compared to TEL-Syk GFP^-^ cells, or both GFP^+^ and GFP^-^ cells from vector expressing mice (Figure S2A). A similar increase in total phospho-tyrosine was also seen in GFP^+^ fetal liver hematopoietic cells retrovirally transduced with TEL-Syk, compared to GFP^-^ cells or vector, Syk and TEL-Syk KD transduced cells (Figure S2C).

**Figure 8:**
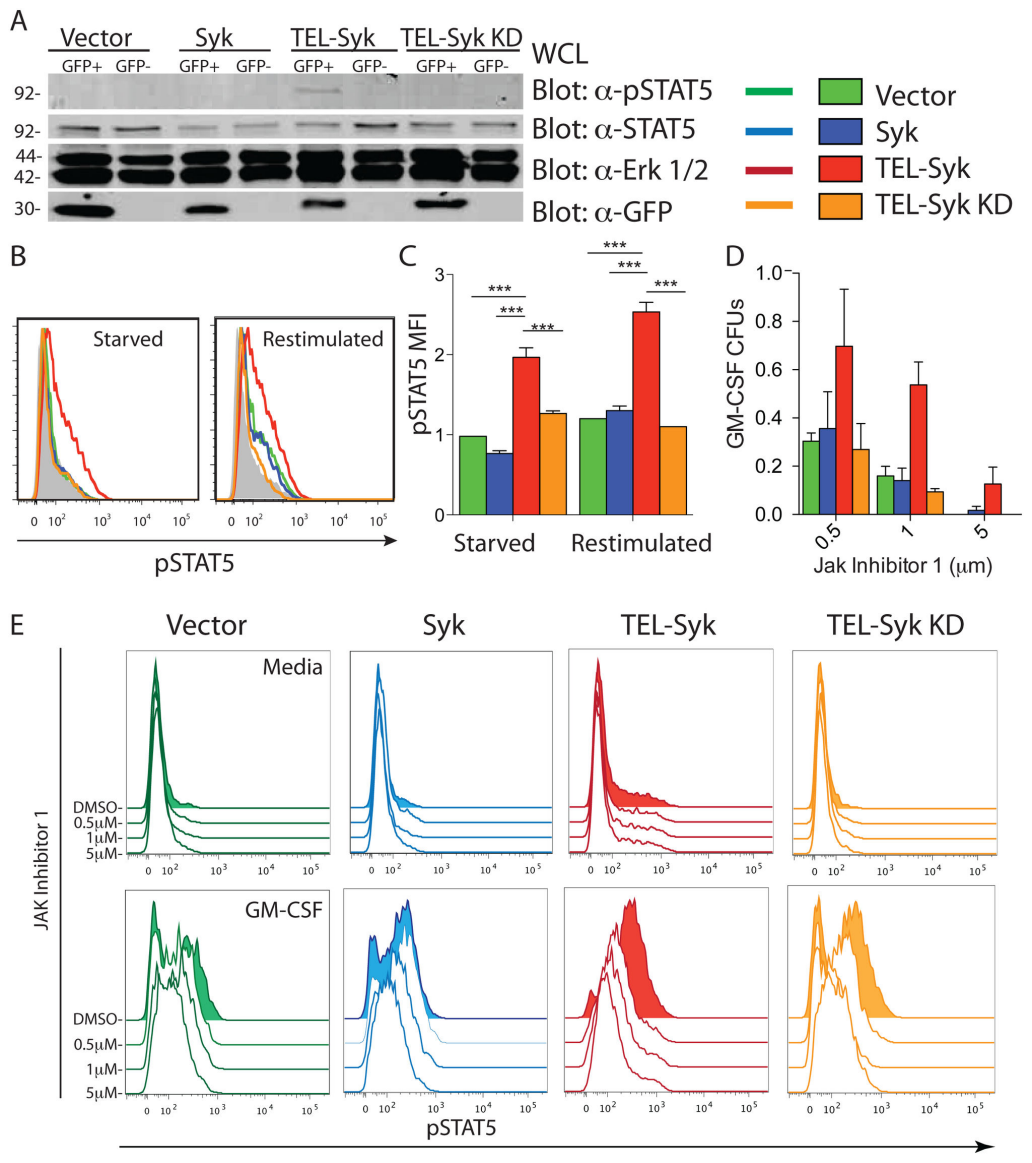
Expression of TEL-Syk led to increased STAT5 phosphorylation and promoted colony formation independent of JAK2 activity. (A) Lysates of sorted GFP^+^ and GFP^-^ fetal liver cells were separated by SDS-PAGE and immunoblotted with indicated antibodies. (B) Representative histograms of retrovirally transduced fetal liver cells cultured for 3 days in cytokines (IL-3, IL-6, SCF), starved for 6 hours, then restimulated with these cytokines. Cells were fixed, permeabilized and levels of phospho-STAT5 in the GFP^+^ cells was determined by flow cytometry. (C) Normalized MFI of phospho-STAT5 in retrovirally transduced fetal liver cultured for 3 days in cytokines, followed by a 6 hour starvation and 30 minute restimulation. Phospho-STAT5 MFIs were normalized to vector expressing cells. (D) Colony formation of sorted GFP+ fetal liver cells in methylcellulose in the presence of a JAK2 inhibitor. Colony numbers were normalized to a DMSO-treated (ie no inhibitor) control for each cell type and represented as a percent of maximal growth after 7 days in culture in 10 ng/mL GM-CSF. (E) Histograms of sorted GFP^+^ fetal liver cells starved for 6 hours and restimulated for 30 minutes with or without 50 ng/mL GM-CSF in the presence of the JAK inhibitor 1 at the indicated concentrations, stained for phospho-STAT5 and assessed by flow cytometry. Experiments were conducted in triplicate. Statistical significance was determined by one-way ANOVA. ***P< 0.001. Data is representative of 3 independent experiments.

To determine whether STAT5 was phosphorylated in TEL-Syk expressing cells, we examined phospho-STAT5 levels in fetal liver cells by immunoblot analysis (Figure 8A) and intracellular staining (Figure 8B). The GFP^+^ TEL-Syk infected cells showed high levels of STAT5 phosphorylation compared to controls, which was apparent even in cells that were cytokine/growth factor starved for 6 hours prior to analysis (Figure 8C). Restimulation with cytokines enhanced phosphorylation of STAT5 in GFP^+^ TEL-Syk and control fetal liver cells (Figure 8B and C).

To determine whether the high level of phospho-STAT5 in TEL-Syk expressing cells was due to activation of the upstream kinase JAK2, we examined colony formation and STAT5 phosphorylation in the presence or absence of a JAK2 inhibitor. For these experiments, we used GM-CSF stimulation, which signals through JAK2 and STAT5 to drive proliferation of hematopoietic cells [20]. As shown in Figure 8D, we found that the JAK2 inhibitor reduced colony formation of fetal liver cells transduced with vector, Syk or TEL-Syk KD in a dose dependent manner compared to DMSO treated controls, while fetal liver hematopoietic cells transduced with TEL-Syk were more resistant to JAK2 inhibition. At high levels of the JAK2 inhibitor vector, Syk and TEL-Syk KD failed to form colonies, while TEL-Syk drove modest colony formation. Using intracellular staining/flow cytometry, we found that TEL-Syk expressing fetal liver hematopoietic cells showed readily detectable levels of phospho-STAT5, in the absence of GM-CSF, which was not affected by increasing doses of the JAK2 inhibitor (Figure 8E, upper panels). Stimulation of these cells with GM-CSF lead to high levels of phospho-STAT5 in all cell types, but was highest in the TEL-Syk transduced cells. STAT5 phosphorylation was reduced to baseline by exposure to 5 µM JAK2 inhibitor in vector, Syk and TEL-Syk KD expressing cells, but was still present, at a level approximately equivalent to unstimulated cells, in TEL-Syk transduced fetal liver cells (Figure 8E, lower panels). These data indicate that TEL-Syk expression leads to significant STAT5 phosphorylation in GM-CSF starved cells, which is independent of JAK2, and very high levels of phopho-STAT5 in GM-CSF stimulated cells, which is mediated predominately through JAK2. The deregulated signaling through STAT5 in TEL-Syk expressing progenitors likely contributes to the myeloproliferation and dysplasia found in TEL-Syk fetal liver chimeric mice. 

## Discussion

In this study, we report that introduction of TEL-Syk into fetal liver hematopoietic cells leads to a rapidly progressive myelodysplasia with dramatic splenic and bone marrow fibrosis. Expression of TEL-Syk in progenitor cells induced a rapid (within 30 days following fetal liver cell transfer) expansion in the number of myeloid cells (neutrophils, monocytes, and eosinophils) in the peripheral blood as well as significant splenic and hepatic extramedullary hematopoiesis. However, with time (by 60 days following fetal liver cell transfer) TEL-Syk chimeric mice developed dramatic bone marrow, splenic and hepatic fibrosis that correlated with the appearance of anemia and thrombocytopenia, which likely contributed to the mortality seen in these animals. The expression of TEL-Syk in progenitors also lead to significant hematopoietic cell apoptosis, as shown by increased cleaved caspase-3 staining, which in combination with the fibrosis was likely the cause of the bone marrow and splenic hypocellularity in older (60 days) chimeras. TEL-Syk mice showed elevated inflammatory cytokines in serum with increases in MMPs, IGFBPs and other angiogenic-related factors. We further demonstrated that fetal liver hematopoietic cells expressing TEL-Syk manifest elevated levels of STAT5 phosphorylation in both resting and cytokine stimulated cells, which was partially resistant to JAK2 inhibition.

Expression of TEL-Syk in fetal liver progenitor cells induces colony formation and proliferation at very low cytokine levels (which otherwise do not support normal progenitor proliferation) due to hyperactivation of cytokine signaling pathways such as JAK2/STAT5. Besides being hyperresponsive to proliferation-inducing cytokines, we found that expression of TEL-Syk leads to overproduction of a number of proinflammatory cytokines. It is likely that cytokine overproduction establishes a paracrine feedback loop that contributes to myeloid cell proliferation and dysplasia in TEL-Syk expressing cells. In other words, both overproduction and hypersensitivity to growth-promoting cytokines could contribute to the MDS caused by TEL-Syk expression in progenitors. The cytokine hypersensitivity also caused skewing of myeloid cell development in *in vitro* assays with increased numbers of abnormal appearing CFU-M colonies arising from Syk-deficient fetal liver cells. Surprisingly, at least in *in vitro* liquid culture assays, we did not observe a significant difference in the proliferation rate of TEL-Syk expressing progenitors compared to vector transduced cells (data not shown). Hence, the increased cell numbers in the TEL-Syk CFU assays must be due to increased cell survival *in vitro*. Since we observed just the opposite *in vivo* (ie increased apoptosis in spleens of TEL-Syk chimeric mice), the complex developmental affects of TEL-Syk expression in progenitors is only partially reflected in standard methylcellulose CFU assays. Perturbation of hematopoietic progenitor populations has also been demonstrated in a mouse model of BCR-ABL-induced myelodysplasia [21]. Expression of BCR-ABL in hematopoietic stem cells leads to a significant increase in splenic-derived myeloid progenitor populations, which contributes to myeloid cell expansion. In this model, overproduction of the proinflammatory cytokine IL-6 is crucial to maintain the myeloid cell expansion.

While expression of TEL-Syk in fetal liver hematopoietic cells induced rapid myeloproliferation with myleodysplasia, we did not observe outgrowth of blast-like cell types in these mice. Moreover, we were unsuccessful in adoptively transferring the myeloproliferative disease to secondary recipient mice using either irradiated or non-irradiated hosts (data not shown). Therefore it is unlikely that the disease process that we observed represents a myeloid cell malignancy, as is seen in mice receiving BCR-ABL transformed progenitor cells [22]. It is likely that the high level of apoptosis induced in myeloid cells by expression of TEL-Syk prevents establishment of myeloproliferation in secondary recipient mice.

The fact that TEL-Syk expression in fetal liver hematopoietic cells leads to a myeloproliferative disease rather than lymphoid leukemia demonstrates a key difference between our data and experiments conducted by Wossning et al [16]. In that work, the authors introduced TEL-Syk into differentiated pre-B cells, rather than a mixed population of hematopoietic cells, leading to CD19^+^ lymphoid leukemia. The variability in disease phenotype is likely to be context dependent such that TEL-Syk introduced into a mixed population of hematopoietic progenitors yields a myeloid disease, while TEL-Syk introduced into a lymphoid precursor yields a lymphoid leukemia. This effect has also been demonstrated in BCR-ABL^+^ CML [21], in which paracrine factors maintain lineage status, but the genetic lesion drives proliferation by deregulated signaling.

The extremely high rate of apoptosis we observed in TEL-Syk expressing mice is likely a major contributor to the bone marrow and splenic hypocellularity that developed in these animals. Increased hematopoietic cell apoptosis is a clinical characteristic of myelofibrosis associated with myeloproliferation in patients [23]. An increased rate of apoptosis could limit the ability of fetal liver hematopoietic cells expressing TEL-Syk to develop secondary genetic changes which would allow establishment of more long lived myeloproliferation or even leukemia. Indeed, chronic myeloproliferative diseases such as CML are associated with reduced rates of apoptosis often through inhibition of stress responses [5], [24].

Analysis of serum cytokines demonstrated an elevation of proinflammatory cytokines such as MCP-1, IL-13, MIP-1α, IL-6, IP-10, MIG, and TCA in mice receiving TEL-Syk expressing fetal liver hematopoietic cells. Though we found no direct evidence of inflammatory induced tissue damage (such as pneumonitis or glomerulonephritis as is seen in other mouse models of inflammatory disease [25]), in combination with the anemia and thrombocytopenia, the proinflammatory nature of the MDS in the TEL-Syk chimeras may contribute to their poor survival. Elevated circulating levels of proinflammatory cytokines have been observed in a number of MPNs in humans. Patients with primary myelofibrosis, with or without the presence of *JAK2V617F*, develop a proinflammatory cytokine signature that contains IL-6, MCP-1, MIG, MIP-1α, TNF-α and IP-10 [26]. The pathologic role of these cytokines is undetermined but their increase correlates with disease prognosis, in particular elevation of IL-6, IL-2R, IL-1RA, MIP-1α, MIG, IL-8, IL-12, IP-10 correlate with shortened primary myelofibrosis survival. Furthermore, numerous proinflammatory cytokines were found in the plasma of PV patients, in which IL-1β, -4, -5, -7, -10, -17, EGF, IFNα, TNF-α, GM-CSF, MIP-1α, MIP-1β, and MCP-1 correlated with reduced survival [27]. Finally, patients with MDS also have elevated cytokines, increases in IP-10, IL-7, and IL-6 being poor prognostic factors for survival [24], [28]. MCP-1, MIG, G-CSF, TNF-α, IL-13, -8, -15, IFN-γ, and HGF were also significantly increased. The authors of these studies suggest that the initial myeloproliferation and the presence of inflammatory circulating cytokines are due to an abnormal bone marrow microenvironment propagated by pathogenic myeloid cells.

Induction of myeloproliferation with myelofibrosis has also been linked to expression of other tyrosine kinases fused to TEL. In particular, expression of a TEL-Lyn fusion protein, originally isolated from a patient with idiopathic myelofibrosis [29], in mouse fetal liver hematopoietic progenitors also leads to an aggressive MPD with excessive bone marrow fibrosis, culminating in lethality by 60-90 days following cell transfer [30]. As we observed with TEL-Syk, the kinase inactive version of TEL-Lyn fails to induce hematopoietic progenitor proliferation, indicating the requirement for kinase activity in both models. Similarly, activation of STAT5 is observed in progenitors transduced with TEL-Lyn. TEL fusion proteins with other tyrosine kinases, such as ABL1, ABL2, JAK2, NTRK3, FFGR3, and PDGFRB, have also been associated with various hematologic malignancies [6], [15], though direct comparisons between these fusion proteins in mouse models is not complete. Recent evidence demonstrated that BCR-ABL circumvents JAK2 and drives STAT5 signaling independently of cytokines in the context of CML [31]. Hantschel et al. observed that BCR-ABL induces STAT5 phosphorylation and CML in JAK2-deficient hematopoietic stem cells. Administration of various JAK2 inhibitors failed to block phosphorylation of STAT5 in Ba/F3 cells transduced with BCR-ABL. These results are consistent with our *in vitro* data demonstrating that TEL-Syk drives phosphorylation of STAT5 and colony formation in fetal liver cells even in the presence of JAK inhibitors. Mechanistically, it is evident that these fusions (BCR-ABL, TEL-Lyn, and TEL-Syk) uncouple the JAK2-STAT5 pathway to drive disease progression.

Overall, these results demonstrate that expression of the TEL-Syk fusion protein in fetal liver hematopoietic cells leads to rapid myeloproliferation and fatal myelofibrosis in a mouse model. The extent and the aggressive nature of this disease is unusual and will serve as a useful model for studying deregulated signaling in the context of myeloproliferative neoplasms with myelofibrosis. Future work with this model may allow identification of the soluble factor(s) derived from the proliferating myeloid cells that contribute to the extensive stromal fibrosis. Though many investigators have implicated myeloid cell-derived factors in myelofibrosis none have been defined.

## Supporting Information

Figure S1 **TEL-Syk lead to myeloid cell expansion.** Analysis of peripheral blood by flow cytometry. Graphs show total cell numbers (A) and numbers of (B) neutrophils (Ly6G^+^ CD11b^+^), (C) B cells (CD19+), and (D) T-cells (TCRβ^+^) at the indicated days following fetal liver cell transfer. Each data point indicates a single animal. Data are shown as mean ± SEM for vector (n=8), Syk (n=7), TEL-Syk (n=3-7)TEL-Syk KD (n=8). Note that this figure shows absolute numbers, while Figure 2H shows percentages, hence the difference in appearance of CD19+ B cells. (E) Detection of eosinophils (Siglec-F^+^ Ly6G^-^) in peripheral blood from a vector and TEL-Syk chimeric mouse at 30 days post cell transfer. Statistical significance was determined by one-way ANOVA. *P< 0.05, **P<0.01, ***P< 0.001.
(TIF)

Figure S2 **Expression of TEL-Syk expression in bone marrow or fetal liver cells leads to increased induced tyrosine phosphorylation.** (A) Immunoblots of 5x10^5^ sorted GFP^+^ and GFP^-^ bone marrow cells from vector, TEL-Syk and TEL-Syk KD chimeric mice were stained with the indicated antibodies. (B) Detection of TEL-Syk transcripts by RT-PCR from 1x10^5^ sorted GFP^+^ and GFP^-^ bone marrow cells from vector, TEL-Syk and TEL-Syk KD chimeric mice. The level of GAPDH was used as a control. (C) Immunoblots of 5x10^5^ sorted GFP^+^ and GFP^-^ from vector, Syk, TEL-Syk and TEL-Syk KD retrovirally transduced fetal liver cells were stained with the indicated antibodies.
(TIF)

Figure S3 **TEL-Syk chimeric mice have abnormal red blood cell morphology.** Wright-Giemsa stains of peripheral blood from vector and TEL-Syk transduced mice at 60 days post fetal liver cell transfer. Scale bars correspond to 10 µm.
(TIF)

Figure S4 **Expression of TEL-Syk disrupted splenic architecture and induced dysplasia.** H&E stained sections of spleens from (A) vector or (B D) TEL-Syk expressing mice. Scale bars correspond to 50 µm (A-C), and 10 μm (D).
(TIF)

Figure S5 **TEL-Syk expression induced cellular infiltration and fibrosis in the liver.** H&E stained sections of livers from (A) vector or (B) TEL-Syk expressing mice. Masson’s Trichrome stained sections of liver from (C) vector or (D) TEL-Syk chimeras to indicate fibrosis. Scale bars correspond to 500 µm (A-D).
(TIF)
