# Supplementary figures and images for: Expression of the TEL-Syk Fusion Protein in Hematopoietic Stem Cells Leads to Rapidly Fatal Myelofibrosis in Mice
Source: PLoS One. 2013 Oct 8;8(10):e77542. doi: 10.1371/journal.pone.0077542 (PMC3792906; doi:10.1371/journal.pone.0077542)

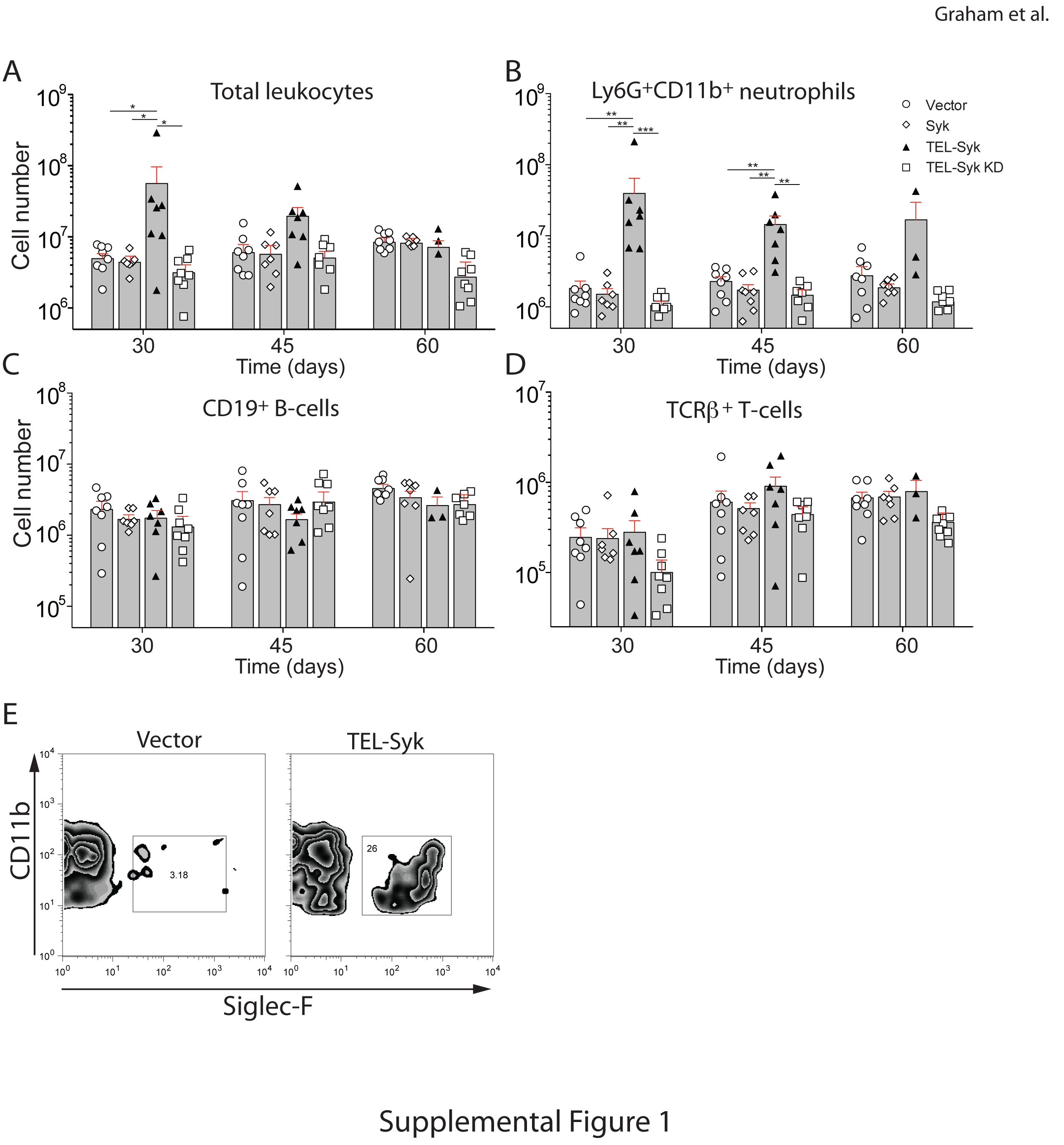

Supplement: Figure S1 — TEL-Syk lead to myeloid cell expansion. Analysis of peripheral blood by flow cytometry. Graphs show total cell numbers (A) and numbers of (B) neutrophils (Ly6G+ CD11b+), (C) B cells (CD19+), and (D) T-cells (TCRβ+) at the indicated days following fetal liver cell transfer. Each data point indicates a single animal. Data are shown as mean ± SEM for vector (n=8), Syk (n=7), TEL-Syk (n=3-7)TEL-Syk KD (n=8). Note that this figure shows absolute numbers, while Figure 2H shows percentages, hence the difference in appearance of CD19+ B cells. (E) Detection of eosinophils (Siglec-F+ Ly6G-) in peripheral blood from a vector and TEL-Syk chimeric mouse at 30 days post cell transfer. Statistical significance was determined by one-way ANOVA. *P< 0.05, **P<0.01, ***P< 0.001. (TIF) [file pone.0077542.s001.tif]

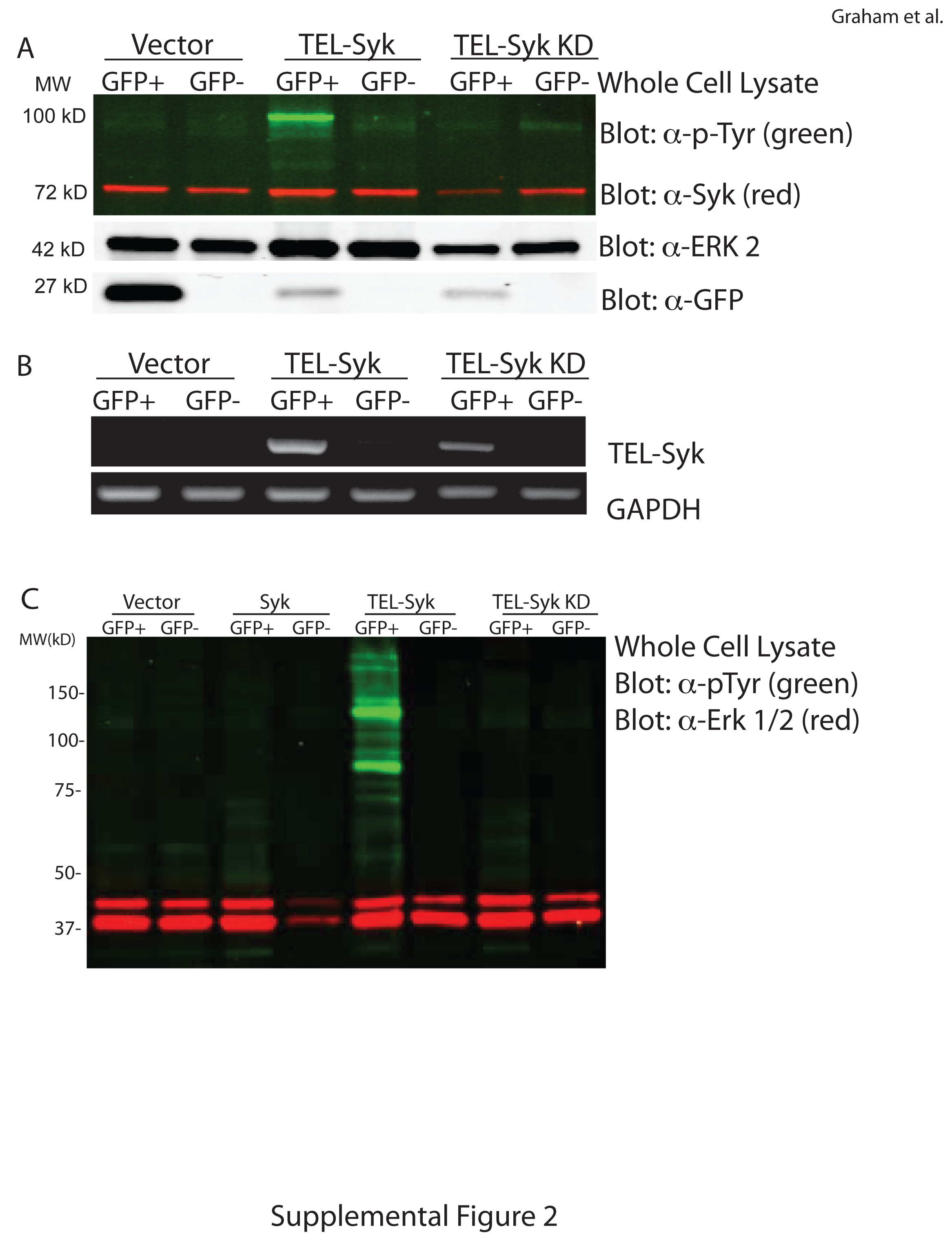

Supplement: Figure S2 — Expression of TEL-Syk expression in bone marrow or fetal liver cells leads to increased induced tyrosine phosphorylation. (A) Immunoblots of 5x10^5^ sorted GFP+ and GFP- bone marrow cells from vector, TEL-Syk and TEL-Syk KD chimeric mice were stained with the indicated antibodies. (B) Detection of TEL-Syk transcripts by RT-PCR from 1x10^5^ sorted GFP+ and GFP- bone marrow cells from vector, TEL-Syk and TEL-Syk KD chimeric mice. The level of GAPDH was used as a control. (C) Immunoblots of 5x10^5^ sorted GFP+ and GFP- from vector, Syk, TEL-Syk and TEL-Syk KD retrovirally transduced fetal liver cells were stained with the indicated antibodies. (TIF) [file pone.0077542.s002.tif]

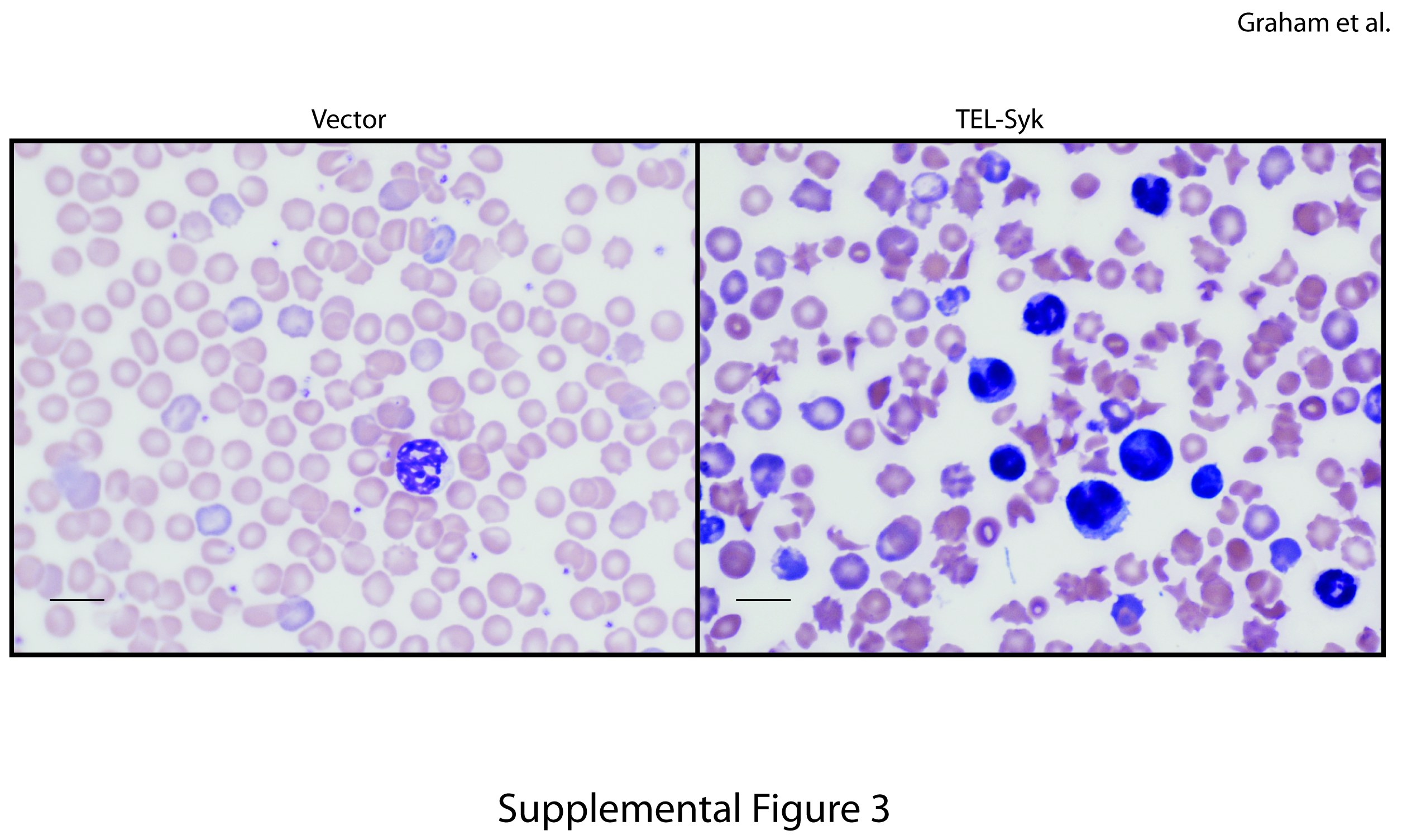

Supplement: Figure S3 — TEL-Syk chimeric mice have abnormal red blood cell morphology. Wright-Giemsa stains of peripheral blood from vector and TEL-Syk transduced mice at 60 days post fetal liver cell transfer. Scale bars correspond to 10 µm. (TIF) [file pone.0077542.s003.tif]

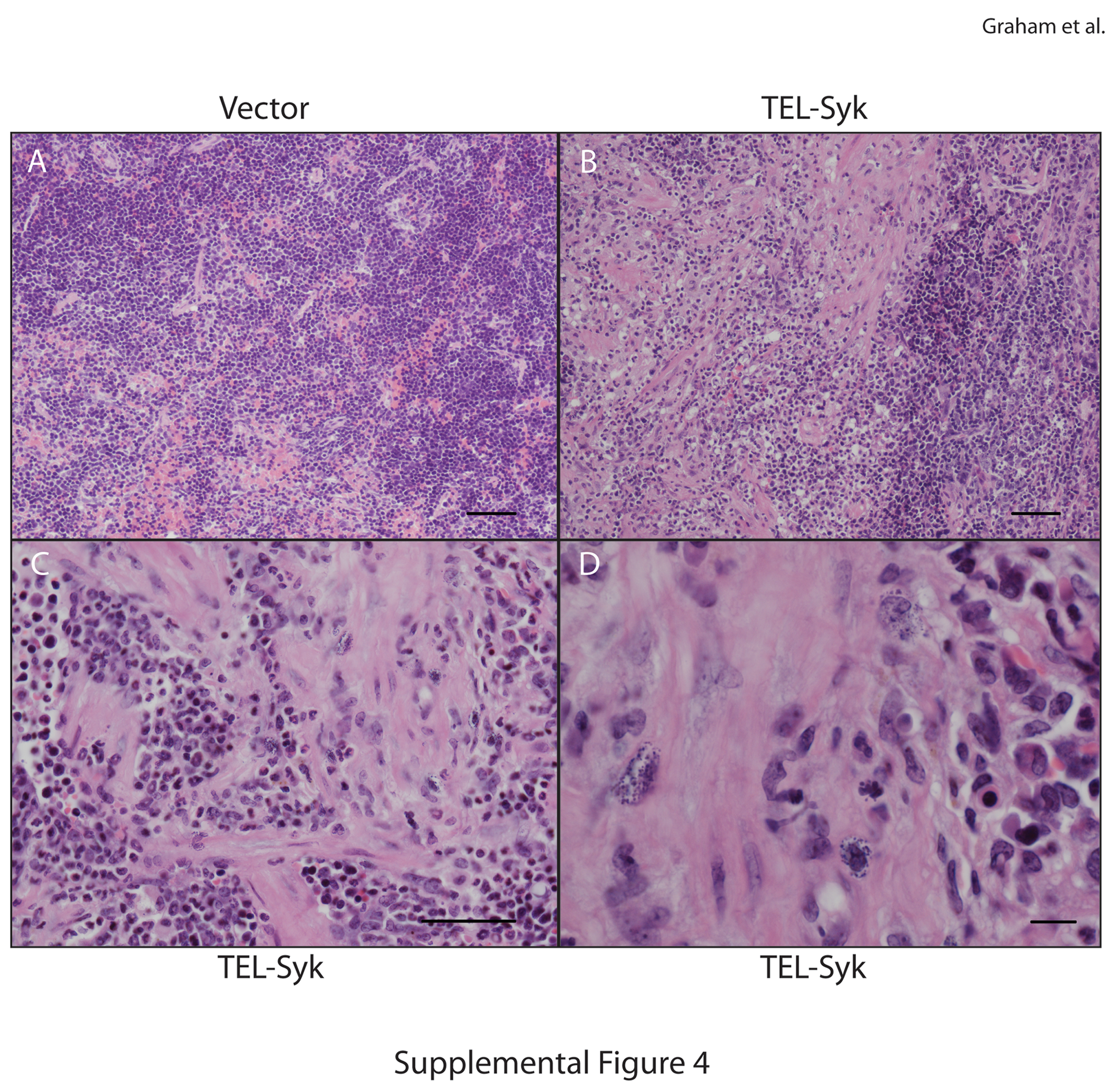

Supplement: Figure S4 — Expression of TEL-Syk disrupted splenic architecture and induced dysplasia. H&E stained sections of spleens from (A) vector or (B D) TEL-Syk expressing mice. Scale bars correspond to 50 µm (A-C), and 10 μm (D). (TIF) [file pone.0077542.s004.tif]

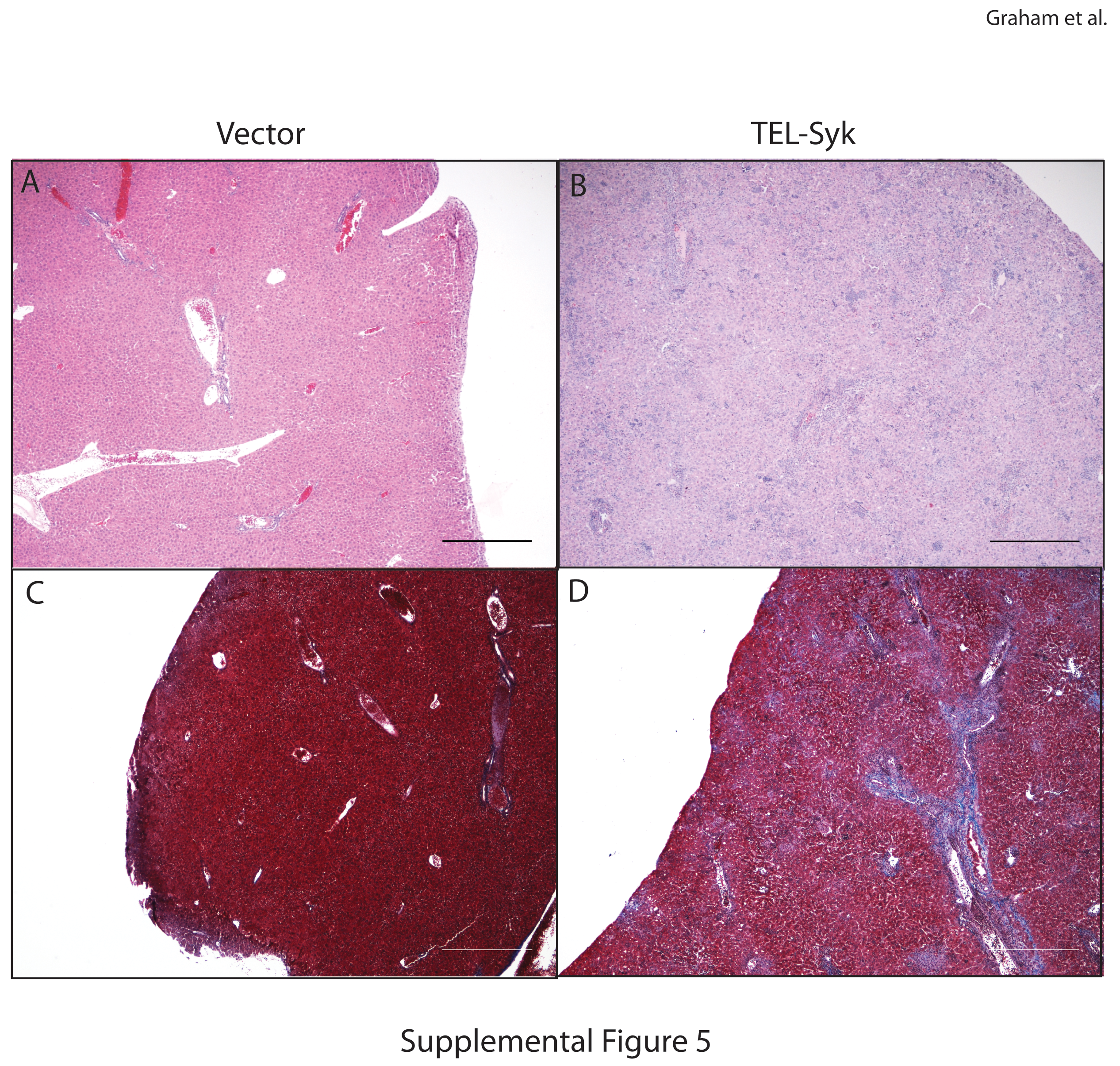

Supplement: Figure S5 — TEL-Syk expression induced cellular infiltration and fibrosis in the liver. H&E stained sections of livers from (A) vector or (B) TEL-Syk expressing mice. Masson’s Trichrome stained sections of liver from (C) vector or (D) TEL-Syk chimeras to indicate fibrosis. Scale bars correspond to 500 µm (A-D). (TIF) [file pone.0077542.s005.tif]
